# Convergence and adaptive discretization of the IRGNM Tikhonov and the IRGNM Ivanov method under a tangential cone condition in Banach space

**DOI:** 10.1007/s00211-018-0971-5

**Published:** 2018-05-29

**Authors:** Barbara Kaltenbacher, Mario Luiz Previatti de Souza

**Affiliations:** Institute of Mathematics, Alpen-Adria-Universität Klagenfurt, Klagenfurt, Austria

**Keywords:** 65F22, 65N20

## Abstract

In this paper we consider the iteratively regularized Gauss–Newton method (IRGNM) in its classical Tikhonov version as well as two further—Ivanov type and Morozov type—versions. In these two alternative versions, regularization is achieved by imposing bounds on the solution or by minimizing some regularization functional under a constraint on the data misfit, respectively. We do so in a general Banach space setting and under a tangential cone condition, while convergence (without source conditions, thus without rates) has so far only been proven under stronger restrictions on the nonlinearity of the operator and/or on the spaces. Moreover, we provide a convergence result for the discretized problem with an appropriate control on the error and show how to provide the required error bounds by goal oriented weighted dual residual estimators. The results are illustrated for an inverse source problem for a nonlinear elliptic boundary value problem, for the cases of a measure valued and of an $L^\infty$ source. For the latter, we also provide numerical results with the Ivanov type IRGNM.

## Introduction

In this paper we consider a nonlinear ill-posed operator equation

1 $$\begin{aligned} F(x)=y\,, \end{aligned}$$

where the possibly nonlinear operator $F:\mathcal {D}(F)\subseteq X \rightarrow Y$ with domain $\mathcal {D}(F)$ maps between real Banach spaces *X* and *Y*. We are interested in the ill-posed situation, i.e., *F* fails to be continuously invertible, and the data are contaminated with noise, thus regularization has to be applied (see, e.g., [8, 27], and references therein).

Throughout this paper we will assume that an exact solution $x^\dagger \in \mathcal {D}(F)$ of (1) exists, i.e., $F(x^\dagger )=y$, and that the noise level $\delta$ in the (deterministic) estimate

2 $$\begin{aligned} \Vert y-y^\delta \Vert \le \delta \end{aligned}$$

is known.

Partially we will also refer to the formulation of the inverse problem as a system of model and observation equation

3 $$\begin{aligned} A(x,u)= & {} 0 \end{aligned}$$

4 $$\begin{aligned} C(u)= & {} y\,. \end{aligned}$$

Here $A:X\times V\rightarrow W^*$ and $C:V\rightarrow Y$ are the model and observation operator, so that with the parameter-to-state map $S:X\rightarrow V$ satisfying $A(x,S(x))=0$ and $F=C\circ S$, (1) is equivalent to the all-at-once formulation (3), (4).

Newton type methods for the solution of nonlinear ill-posed problems (1) have been extensively studied in Hilbert spaces (see, e.g., [2, 20] and the references therein) and more recently also in a in Banach space setting. In particular, the iteratively regularized Gauss–Newton method [1] can be generalized to a Banach space setting by calculating iterates $x_{k+1}^{\delta }$ in a Tikhonov type variational form as

5 $$\begin{aligned} x_{k+1}^\delta \in \mathrm{argmin}_{x\in \mathcal {C}} \ \Vert F'(x_k^\delta )(x-x_k^\delta )+F(x_k^\delta )-y^\delta \Vert ^p +\alpha _k\mathcal {R}(x)\,, \end{aligned}$$

see, e.g., [11, 16, 17, 21, 28] where $p\in [1,\infty )$, $(\alpha _k)_{k\in \mathbb {N}}$ is a sequence of regularization parameters, and $\mathcal {R}$ is some nonnegative regularization functional. Alternatively, one might introduce regularization by imposing some bound $\rho _k$ on the norm of *x*, or, again, generally, on a regularization functional of *x*

6 $$\begin{aligned} x_{k+1}^{\delta } \in \mathrm{argmin}_{ x\in \mathcal {C} } \ \Vert F'(x_k^\delta )(x-x_k^\delta )+F(x_k^\delta )-y^\delta \Vert \text{ such } \text{ that } \mathcal {R}(x)\le \rho _k\,, \end{aligned}$$

which corresponds to Ivanov regularization or the method of quasi solutions, see, e.g., [7, 13–15, 22, 24, 26]. A third way of incorporating regularization in a Newton type iteration is Morozov regularization, also called the method of the residuals, see, e.g., [9, 22, 23]

7 $$\begin{aligned} x_{k+1}^{\delta } \in \mathrm{argmin}_{ x\in \mathcal {C} } \ \mathcal {R}(x) \text{ such } \text{ that } \Vert F'(x_k^\delta )(x-x_k^\delta )+F(x_k^\delta )-y^\delta \Vert \le \sigma \Vert F(x_k^\delta )-y^\delta \Vert \,, \end{aligned}$$

for some $\sigma \in (0,1)$, where the choice of the bound in the inequality constraint is very much inspired by the inexact Newton type regularization parameter choice in [10].

We restrict ourselves to the norm in *Y* as a measure of the data misfit, but the analysis could as well be extended to more general functionals $\mathcal {S}$ satisfying certain conditions, as e.g., in [11, 28]. Here $\mathcal {C}$ is a set (possibly chosen with convenient properties for carrying out the minimization) containing $x^\dagger$ and being contained in $\mathcal {D}(F)$, such that *F* satisfies additional conditions on $\mathcal {C}$ see (8), (10) below. If *F* is defined on all of *X*, then the minimization problem (5) can be posed in an unconstrained way $\mathcal {C}=X$.

As a restriction on the nonlinearity of the forward operator *F* we impose the tangential cone condition

8 $$\begin{aligned} \Vert F(\tilde{x})-F(x)-F'(x)(\tilde{x}-x)\Vert \le c_{tc}\Vert F(\tilde{x})-F(x)\Vert \text{ for } \text{ all } \tilde{x},x\in \mathcal {B}_R \end{aligned}$$

(also called Scherzer condition, cf. [25]) for some constant $c_{tc}<1/3$. Here, for any $r>0$,

9 $$\begin{aligned} \mathcal {B}_r =\{ x\in \mathcal {C}\, : \, \mathcal {R}(x)\le r\} \end{aligned}$$

is a sublevel set of the regularization functional and *R* will be specified in the convergence result Theorem 1.

Note that the convergence conditions imposed in [11, 16, 17, 21, 28] in the situation without source condition, namely local invariance of the range of $F'(x)^*$, are slightly stronger, since this adjoint range invariance is sufficient for (8). However, most probably the gap is not very large, as in those application examples where (8) has been verified, the proof of (8) is actually often done via adjoint range invariance. In (5), (6), (7), the bounded linear operator $F'(x)$ is not necessarily a Gâteaux or Fréchet derivative of *F*, but just some local linearization (in the sense of (8)), satisfying additionally the weak closedness condition

10 $$\begin{aligned}&\forall x\in \mathcal {C}\,, \ (x_n)_{n\in \mathbb {N}}\subseteq \mathcal {C}\, : \ \Bigl (x_n{\mathop {\longrightarrow }\limits ^{\mathcal {T}_X}} \hat{x}\,, \text{ and } F'(x)x_n{\mathop {\longrightarrow }\limits ^{\mathcal {T}_Y}} y \Bigr ) \nonumber \\&\quad \Rightarrow \ \Bigl (\hat{x}\in \mathcal {C} \text{ and } F'(x)\hat{x}=y\Bigr )\,. \end{aligned}$$

In here, $\mathcal {T}_X$ and $\mathcal {T}_Y$ are topologies on *X* and *Y* (e.g., just the weak or weak* topologies) such that bounded sets in *Y* are $\mathcal {T}_Y$-compact and the norm in *Y* is $\mathcal {T}_Y$-lower semicontinuous.

The remainder of this paper is organized as follows. In Sect. 2 we state and prove convergence results in the continuous and discretized setting. Section 3 shows how to actually obtain the required discretization error estimates by a goal oriented weighted dual residual approach and Sect. 4 illustrates the theoretical findings by an inverse souce problem for a nonlinear PDE. In Sect. 5 we provide some numerical results for this model problem and Sect. 6 concludes with some remarks.

## Convergence

In this section we will study convergence of the IRGNM iterates first of all in a continuous setting, then in the situation of having discretized for computational purposes.

The regularization parameters $\alpha _k$, $\rho _k$, $\sigma$ are chosen a priori

11 $$\begin{aligned} \alpha _k=\alpha _0 \theta ^k \text{ for } \text{ some } \theta \in \left( \left( {\textstyle \frac{2c_{ct}}{1-c_{ct}}}\right) ^p,1\right) \end{aligned}$$

(note that $({\textstyle \frac{2c_{ct}}{1-c_{ct}}})^p<1$ for $c_{tc}<1/3$),

12 $$\begin{aligned} \rho _k\equiv \rho \ge \mathcal {R}(x^\dagger )\,, \end{aligned}$$

and

13 $$\begin{aligned} \sigma \ge \frac{1+c_{tc}}{\tau }+c_{tc}\,, \quad \sigma <1-2c_{tc}\,, \end{aligned}$$

with $\tau$ as in (14), and the iteration is stopped according to the discrepancy principle

14 $$\begin{aligned} k_*=k_*(\delta ,y^\delta )=\min \{k\in \mathbb {N}_0\ : \ \Vert F(x_k^{\delta })-y^\delta \Vert \le \tau \delta \} \end{aligned}$$

with some fixed $\tau >1$ chosen sufficiently large but independent of $\delta$.

### Theorem 1

Let $\mathcal {R}:X\rightarrow [0,\infty ]$ be proper, convex and $\mathcal {T}_X$ lower semicontinuous with $\mathcal {R}(x^\dagger )<\infty$ and let, for all $r\in [\mathcal {R}(x^\dagger ),\infty )$ in case of (5), or for all $r\in [\mathcal {R}(x^\dagger ),\rho ]$ in case of (6), or for $r=\mathcal {R}(x^\dagger )$ in case of (7), the sublevel set (9) be compact with respect to the topology $\mathcal {T}_X$ on *X*.

Moroever, let *F* satisfy (8), (10).

Finally, let the family of data $(y^\delta )_{\delta >0}$ satisfy (2).

(i) Then for fixed $\delta$, $y^\delta$, the iterates according to (5)–(7) are well-defined and satisfy
  15 $$\begin{aligned} x_k^\delta \in \mathcal {B}_R \text{ with } R \, \left\{ \begin{array}{l} \text{ defined } \text{ by } \text {{(}23{)}},\, \text {{(}19{)}},\, \text {{(}20{)}} \text{ in } \text{ case } \text{ of } {(}5{)}\\ =\rho \text{ in } \text{ case } \text{ of } \text {{(}6{)}}\\ =\mathcal {R}(x^\dagger ) \text{ in } \text{ case } \text{ of } \text {{(}7{)}} \end{array}\right. \end{aligned}$$
  for all $k\le k_*(\delta ,y^\delta )$, which denotes the stopping index according to the discrepancy principle (14) with $\tau$ sufficiently large, and this stopping indes $k_*(\delta ,y^\delta )$ is finite.
(ii) Moreover, for both methods we have $\mathcal {T}_X$-subsequential convergence as $\delta \rightarrow 0$ i.e., $(x^\delta _{k_*(\delta ,y^\delta )})_{\delta >0}$ has a $\mathcal {T}_X$-convergent subsequence and the limit of every $\mathcal {T}_X$-convergent subsequence solves (1). If the solution $x^\dagger$ of (1) is unique in $\mathcal {B}_R$, then $x^\delta _{k_*(\delta ,y^\delta )}{\mathop {\longrightarrow }\limits ^{\mathcal {T}_X}}x^\dagger$ as $\delta \rightarrow 0$.
(iii) Additionally, $k_*$ satisfies the asymptotics $k_*=\mathcal {O}(\log (1/\delta ))$.

### Proof

Existence of minimizers $x_{k+1}^\delta$ of (5)–(7) for fixed *k*, $x_k^\delta$ and $y^\delta$ follows by the direct method of calculus of variations: In all three cases, the cost functional

$$\begin{aligned} J_k(x)= & {} \Vert F'(x_k^\delta )(x-x_k^\delta )+F(x_k^\delta )-y^\delta \Vert ^p+\alpha _k\mathcal {R}(x) \text{ in } \text{ case } \text{ of } \text {(5)},\\ J_k(x)= & {} \frac{1}{2}\Vert F'(x_k^\delta )(x-x_k^\delta )+F(x_k^\delta )-y^\delta \Vert ^2 \text{ in } \text{ case } \text{ of } \text {(6)},\\ J_k(x)= & {} \mathcal {R} \text{ in } \text{ case } \text{ of } \text {(7)}, \end{aligned}$$

is bounded from below and the admissible set

$$\begin{aligned} X^{\mathrm{ad}}= & {} \mathcal {C} \text{ in } \text{ case } \text{ of } \text {(5)}, \\ X^{\mathrm{ad}}= & {} \mathcal {B}_{\rho } \text{ in } \text{ case } \text{ of } \text {(6)},\\ X^{\mathrm{ad}}= & {} \{x\in \mathcal {C}\, : \, \Vert F'(x_k^\delta )(x-x_k^\delta )+F(x_k^\delta )-y^\delta \Vert \le \sigma \Vert F(x_k^\delta )-y^\delta \Vert \} \text{ in } \text{ case } \text{ of } \text {(7)} \end{aligned}$$

is nonempty (for (6) this follows from $\rho _k\ge \mathcal {R}(x^\dagger )$ and for (7) from (8), (14) and (13), see (16) below). Hence, there exists a minimizing sequence $(x^l)_{l\in \mathbb {N}}\subseteq X^{\mathrm{ad}}\cap \mathcal {B}_r$ for

$$\begin{aligned} r=\frac{1}{\alpha _k}J_k(x^\dagger ) \text{ in } \text{ case } \text{ of } \text {(5)}, \quad r=\rho \text{ in } \text{ case } \text{ of } \text {(6)}, \quad r=\mathcal {R}(x^\dagger ) \text{ in } \text{ case } \text{ of } \text {(7)}, \end{aligned}$$

with bounded linearized residuals $\Vert F'(x_k^\delta )(x^l-x_k^\delta )+F(x_k^\delta )-y^\delta \Vert \le s$ for

$$\begin{aligned} s=J_k(x^\dagger )^{1/p} \text{ in } \text{ case } \text{ of } \text {(5)},\, \text {(6)}, \quad s=\sigma \Vert F(x_k^\delta )-y^\delta \Vert \text{ in } \text{ case } \text{ of } \text {(7)}, \end{aligned}$$

and $\lim _{l\rightarrow \infty }J_k(x^l)=\inf _{x\in X^{\mathrm{ad}}} J_k(x)$. By $\mathcal {T}_X$-compactness of $\mathcal {B}_r$, the sequence $(x^l)_{l\in \mathbb {N}}$ has a $\mathcal {T}_X$-convergent subsequence $(x^{l_m})_{m\in \mathbb {N}}$ with limit $\bar{x}\in \mathcal {B}_r$. Moroever, $\mathcal {T}_Y$-compactness of norm bounded sets in *Y* together with $\mathcal {T}_X$-$\mathcal {T}_Y$-closedness of $F'(x_k^\delta )$ and lower $\mathcal {T}_Y$ semicontinuity of the norm in *Y*, implies that in all three cases $J_k(\bar{x})\le \liminf _{m\rightarrow \infty }J_k(x^{l_m})=\inf _{x\in X^{\mathrm{ad}}} J_k(x)$ and $\bar{x}\in X^{\mathrm{ad}}$, hence $\bar{x}$ is a minimizer.

Note that (ii) follows from (i) by standard arguments and our assumption on $\mathcal {T}$-compactness of $\mathcal {B}_R$. Thus it remains to prove (i) and (iii) for the three versions (5), (6), (7) of the IRGNM.

For this purpose we are going to show that for every $\delta >0$, there exists $k_*=k_*(\delta ,y^\delta )$ such that $k_* \sim \log (1/\delta )$, and the stopping criterion according to the discrepancy principle $\Vert F(x^\delta _{k_*(\delta ,y^\delta )})-y^\delta \Vert \le \tau \delta$ is satisfied. For (5), we also need to show that $\mathcal {R}(x_k^\delta )\le R$ for $k\le k_*(\delta ,y^\delta )$, whereas in (6) this automatically holds by (12). The same holds true for (7): If $x_k^\delta \in \mathcal {B}_R$, then by (8), (14) and (13) we have

16 $$\begin{aligned}&\Vert F'(x_k^\delta )(x^\dagger -x_k^\delta )+F(x_k^\delta )-y^\delta \Vert \le c_{tc} \Vert F(x_k^\delta )-y^\delta \Vert +(1+c_{tc})\delta \nonumber \\&\quad \le \left( c_{tc}+\frac{1+c_{tc}}{\tau }\right) \Vert F(x_k^\delta )-y^\delta \Vert , \end{aligned}$$

so $x^\dagger$ is admissible, hence $\mathcal {R}(x_{k+1}^\delta )\le \mathcal {R}(x^\dagger )$, i.e., $x_{k+1}^\delta \in \mathcal {B}_R$.

We start with the Tikhonov version (5) and carry out an induction proof of the following statement: For all $k\in \{0,\ldots ,k_*(\delta ,y^\delta )\}$

17 $$\begin{aligned} \mathcal {R}(x_k^\delta )\le R \text{ and } \forall j\in \{0,\ldots ,k-1\}\, : \ d_{j+1}+\alpha _j\mathcal {R}_{j+1}\le qd_j +\alpha _j\mathcal {R}^\dagger +C\delta ^p\,, \end{aligned}$$

where

18 $$\begin{aligned} d_k&:= 2^{1-p}(1-c_{tc})^p\Vert F(x_k^\delta )-y^\delta \Vert ^p, \end{aligned}$$

19 $$\begin{aligned} q&:=2^{p-1}\bigl ((1+\gamma )^{p-1}+1\bigr )\left( \frac{c_{tc}}{1-c_{tc}}\right) ^{p} \in (0,1), \end{aligned}$$

20 $$\begin{aligned} \mathcal {R}_k&:=\mathcal {R}(x_k^\delta ), \quad \mathcal {R}^\dagger :=\mathcal {R}(x^\dagger ),\nonumber \\ C&:=\left( \frac{1+\gamma }{\gamma }\right) ^{p-1}(1+c_{tc})^p, \end{aligned}$$

for some fixed small $\gamma \in (0,1)$. We will require $\frac{q}{\theta }<1$, which by definition of *q* (19) is achievable for $\gamma >0$ sufficiently small, due to $\theta >({\textstyle \frac{2c_{ct}}{1-c_{ct}}})^p$, cf. (11). By Lemma 2 (see the “Appendix”) the right hand side estimate in (17) implies

21 $$\begin{aligned} d_{k}+\alpha _{k-1}\mathcal {R}_{k} < q^{k}d_0 +\left( \frac{1}{1-\frac{q}{\theta }}\right) \alpha _{k-1}\mathcal {R}^\dagger +\left( \frac{1}{1-q}\right) C\delta ^p. \end{aligned}$$

Using the minimality of $x_{k+1}^\delta$ and (2), (8) together with $x^\dagger ,x_k^\delta \in \mathcal {B}_R$, we have

22 $$\begin{aligned}&\Vert F'(x_k^\delta )(x_{k+1}^\delta -x_k^\delta )+F(x_k^\delta )-y^\delta \Vert ^p +\alpha _k\mathcal {R}(x_{k+1}^\delta ) \nonumber \\&\quad \le \Vert F'(x_k^\delta )(x^\dagger -x_k^\delta )+F(x_k^\delta )-y^\delta \Vert ^p + \alpha _k\mathcal {R}(x^\dagger ) \nonumber \\&\quad \le \Bigl (c_{tc}\Vert F(x_k^\delta )-y^\delta \Vert +(1+c_{tc})\delta \Bigr )^p + \alpha _k\mathcal {R}(x^\dagger ), \end{aligned}$$

From (21) and (14) we infer

$$\begin{aligned} \left( 2^{1-p}(1-c_{tc})^p-\frac{C}{(1-q)\tau ^p}\right) \Vert F(x_k^\delta )-y^\delta \Vert ^p\le q^{k}d_0 +\theta ^k\alpha _0\frac{\mathcal {R}^\dagger }{\theta -q} \end{aligned}$$

Using this and again (14) in (22) yields

23 $$\begin{aligned} \mathcal {R}(x_{k+1}^\delta )\le & {} \left( c_{ct}+\frac{1+c_{tc}}{\tau }\right) ^p \left( 2^{1-p}(1-c_{tc})^p-\frac{C}{(1-q)\tau ^p}\right) ^{-1}\nonumber \\&\times \left( \left( \frac{q}{\theta }\right) ^{k}\frac{d_0}{\alpha _0} +\frac{\mathcal {R}^\dagger }{\theta -q}\right) +\mathcal {R}^\dagger \nonumber \\\le & {} \left( \frac{1}{3}+\frac{4}{3\tau }\right) ^p \left( \frac{2}{3^p}-\frac{C}{(1-q)\tau ^p}\right) ^{-1}\nonumber \\&\times \left( 2^{1-p}\frac{\Vert F(x_0^\delta )-y^\delta \Vert ^p}{\alpha _0} +\frac{\mathcal {R}^\dagger }{\theta -q}\right) +\mathcal {R}^\dagger =:R\,. \end{aligned}$$

On the other hand, since we have established $x_{k+1}^\delta \in \mathcal {B}_R$, we can apply (8) to the left hand side of (22) to obtain

$$\begin{aligned}&\Vert F'(x_k^\delta )(x_{k+1}^\delta -x_k^\delta )+F(x_k^\delta )-y^\delta \Vert ^p +\alpha _k\mathcal {R}(x_{k+1}^\delta ) \\&\quad \ge \Bigl | (1-c_{tc})\Vert F(x_{k+1}^\delta )-y^\delta \Vert -c_{tc}\Vert F(x_k^\delta )-y^\delta \Vert \Bigr |^p +\alpha _k\mathcal {R}(x_{k+1}^\delta ). \end{aligned}$$

see also [11, Lemma 5.2] and [21, proof of Theorem 3].

To handle the power *p* we make use of the following inequalities that can be proven by solving extremal value problems, see the “Appendix”

24 $$\begin{aligned} (a+b)^p\le & {} (1+\gamma )^{p-1}a^p+\left( \frac{1+\gamma }{\gamma }\right) ^{p-1}b^p \text{ and } \nonumber \\ (a-b)^p\ge & {} (1-\epsilon )^{p-1}a^p-\left( \frac{1-\epsilon }{\epsilon }\right) ^{p-1}b^p, \end{aligned}$$

for all $a,b > 0,$ $p \ge 1$ and $\gamma , \epsilon \in (0,1)$, where for the right hand inequality to hold, additionally $a\ge b$ is needed.

Hence, in case $(1-c_{tc})\Vert F(x_{k+1}^\delta )-y^\delta \Vert \ge c_{tc}\Vert F(x_k^\delta )-y^\delta \Vert$ the following general estimate holds

25 $$\begin{aligned}&(1-\epsilon )^{p-1}(1-c_{tc})^p\Vert F(x_{k+1}^\delta )-y^\delta \Vert ^p+\alpha _k\mathcal {R}(x_{k+1}^\delta )\nonumber \\&\quad \le \left( (1+\gamma )^{p-1}+\left( \frac{1-\epsilon }{\epsilon }\right) ^{p-1}\right) c_{tc}^p\Vert F(x_k^\delta )-y^\delta \Vert ^p\nonumber \\&\qquad +\,\alpha _k\mathcal {R}(x^\dagger )+\left( \frac{1+\gamma }{\gamma }\right) ^{p-1}(1+c_{tc})^p\delta ^p, \end{aligned}$$

for $$\gamma , \epsilon \in (0,1).$$

So in order for this recursion to yield geometric decay of $\Vert F(x_k^\delta )-y^\delta \Vert$, we need to ensure

26 $$\begin{aligned} (1-\epsilon )^{p-1}(1-c_{tc})^p > \left( (1+\gamma )^{p-1}+\left( \frac{1-\epsilon }{\epsilon }\right) ^{p-1}\right) c_{tc}^p \end{aligned}$$

for a proper choice of $\epsilon ,\gamma \in (0,1)$. To obtain the largest possible (and therefore least restrictive) bound on $c_{tc}$, we rewrite the requirement above as

$$\begin{aligned} \left( \frac{c_{tc}}{1-c_{tc}}\right) ^p< & {} \sup _{\epsilon ,\gamma \in (0,1)} (1-\epsilon )^{p-1}\left( (1+\gamma )^{p-1}+\left( \frac{1-\epsilon }{\epsilon }\right) ^{p-1}\right) ^{-1}\\= & {} \sup _{\epsilon \in (0,1)} \underbrace{(1-\epsilon )^{p-1}\left( 1+\left( \frac{1-\epsilon }{\epsilon }\right) ^{p-1}\right) ^{-1}}_{=\phi (\epsilon )} = \phi ({\textstyle \frac{1}{2}})=2^{-p}, \end{aligned}$$

as can be found out by evaluating the derivative of $\phi$

$$\begin{aligned} \phi '(\epsilon )= -(p-1)(1-\epsilon )^{p-2}\left( 1+\left( \frac{1-\epsilon }{\epsilon }\right) ^{p-1}\right) ^{-2}\left( 1-\left( \frac{1-\epsilon }{\epsilon }\right) ^p\right) \,. \end{aligned}$$

Thus we will furtheron set $\epsilon =\frac{1}{2}$ and assume that $\gamma >0$ is sufficiently small so that (26) holds with $\epsilon =\frac{1}{2}$, i.e., (19). Then, using (11), estimate (25) can be written as

27 $$\begin{aligned} d_{k+1}+\alpha _k\mathcal {R}_{k+1}\le qd_k +\alpha _0\theta ^k\mathcal {R}^\dagger +C\delta ^p, \end{aligned}$$

which we first of all regard as a recursive estimate for $d_k$.

To derive a similar estimate also in the complementary case $(1-c_{tc})\Vert F(x_{k+1}^\delta )-y^\delta \Vert < c_{tc}\Vert F(x_k^\delta )-y^\delta \Vert$, we use that fact that, for $d_k$ as in (18), this inequality just means

$$\begin{aligned} d_{k+1}<\left( \frac{c_{tc}}{1-c_{tc}}\right) ^p d_k \end{aligned}$$

and, using (22) and the left hand part of (24),

$$\begin{aligned} \alpha _k\mathcal {R}_{k+1}\le (1+\gamma )^{p-1}c_{tc}^p\Vert F(x_k^\delta )-y^\delta \Vert ^p+\alpha _k\mathcal {R}^\dagger +\left( \frac{1+\gamma }{\gamma }\right) ^{p-1}(1+c_{tc})^p\delta ^p, \end{aligned}$$

hence after addition we again get (27) (even with a slightly smaller value of $q:=(1+2^{p-1}(1+\gamma )^{p-1})(\frac{c_{tc}}{1-c_{tc}})^{p}$).

Thus in both cases, using Lemma 2 we can conclude that

28 $$\begin{aligned} d_{k+1}+\alpha _k\mathcal {R}_{k+1} < q^{k+1}d_0 +\left( \frac{1}{1-\frac{q}{\theta }}\right) \alpha _k\mathcal {R}^\dagger +\left( \frac{1}{1-q}\right) C\delta ^p. \end{aligned}$$

This finishes the induction proof of (17) for all $k\in \{0,\ldots ,k_*(\delta ,y^\delta )\}$.

We next show that the discrepancy stopping criterion from (14), i.e., $d_{k_*} \le \tilde{\tau }\delta ^p$ for $\tilde{\tau }=2^{1-p}(1-c_{tc})^p\tau ^p$, will be satisfied after finitely many, namely $O(\log (1/\delta ))$, steps. For this purpose, note that $\tilde{\tau }>\frac{C}{1-q}$, provided $\tau$ is chosen sufficiently large, which we assume to be done. Thus, indeed, using (11), (21), we have

29 $$\begin{aligned} d_{k}\le d_{k}+\alpha _{k-1}\mathcal {R}_{k} < \theta ^{k}\Bigl (d_0 +\frac{\alpha _0}{\theta -q} \mathcal {R}^\dagger \Bigr )+\frac{C}{1-q}\delta ^p, \end{aligned}$$

where the right hand side falls below $\tilde{\tau }\delta ^p$ as soon as

$$\begin{aligned} k&\ge (\log 1/\theta )^{-1}\left( p\log (1/\delta ) +\log \left( d_0+\frac{\alpha _0}{\theta -q}\mathcal {R}^\dagger \right) -\log \left( \tilde{\tau }-\frac{C}{1-q}\right) \right) \\&=:\bar{k}(\delta ). \end{aligned}$$

Thus we get the upper estimate $k_{*}(\delta ,y^\delta )\le \bar{k}(\delta )=O(\log (1/\delta ))$.

For the Ivanov version (6), it only remains to show finiteness of the stopping index, as boundedness of the $\mathcal {R}$ values by $R=\rho$ holds by definition. Applying the minimality argument with $x^\dagger$ being admissible [cf. (12)] to (6) leads to the special case $p=1$, $\alpha _k=0$ in (25)

$$\begin{aligned} (1-c_{tc})\Vert F(x_{k+1}^\delta )-y^\delta \Vert \le 2c_{tc}\Vert F(x_k^\delta )-y^\delta \Vert +(1+c_{tc})\delta . \end{aligned}$$

Our notation becomes

$$\begin{aligned} d_k&:= (1-c_{tc})\Vert F(x_k^\delta )-y^\delta \Vert ,\\ q&:=\frac{2c_{tc}}{1-c_{tc}} \in (0,1),\\ C&:=(1+c_{tc}), \end{aligned}$$

which gives

$$\begin{aligned} d_{k+1} \le qd_k+C\delta , \end{aligned}$$

and by induction, one can conclude

$$\begin{aligned} d_{k} < q^{k}d_0 +\left( \frac{1}{1-q}\right) C\delta , \end{aligned}$$

where the right hand side is smaller than $\tilde{\tau }\delta$ (with $\tilde{\tau }=(1-c_{tc})\tau$) for all

$$\begin{aligned} k \ge (\log 1/q)^{-1}\left( p\log (1/\delta ) +\log d_0 -\log \left( \tilde{\tau }-\frac{C}{1-q}\right) \right) =:\bar{k}(\delta ), \end{aligned}$$

so that we can again conclude $k_{*}(\delta ,y^\delta )\le \bar{k}(\delta )=O(\log (1/\delta ))$.

Finally we consider (7), where boundedness of the $\mathcal {R}$ values by $R=\mathcal {R}(x^\dagger )$ holds by minimality and the fact that $x^\dagger$ is admissible, cf. (16). Geometric decay of the residuals follows by the estimate

30 $$\begin{aligned} \sigma \Vert F(x_k^\delta )-y^\delta \Vert\ge & {} \Vert F'(x_k^\delta )(x_{k+1}^\delta -x_k^\delta )+F(x_k^\delta )-y^\delta \Vert \nonumber \\\ge & {} (1-c_{tc})\Vert F(x_{k+1}^\delta )-y^\delta \Vert -c_{tc}\Vert F(x_k^\delta )-y^\delta \Vert \end{aligned}$$

and (13), i.e.,

$$\begin{aligned} \Vert F(x_{k+1}^\delta )-y^\delta \Vert \le q \Vert F(x_k^\delta )-y^\delta \Vert \end{aligned}$$

with

$$\begin{aligned} q=\frac{\sigma +c_{tc}}{1-c_{tc}} \,, \end{aligned}$$

so that similarly to above we end up with a logarithmic estimate for $k_*$. $\square$

### Remark 1

Convergence of $\mathcal {R}(x_{k_*(\delta ,y^\delta )}^\delta )$ to $\mathcal {R}(x^\dagger )$ as $\delta \rightarrow 0$ holds along the $\mathcal {T}_X$ convergent subsequence according to Theorem 1 (ii), first of all for the Morozov and the Ivanov version of the IRGNM, with the choice $\rho =\mathcal {R}(x^\dagger )$ for the latter, since in both cases $\mathcal {R}(x_{k_*(\delta ,y^\delta )}^\delta )\le \mathcal {R}(x^\dagger )$ holds for all $\delta$ and $\mathcal {R}$ is $\mathcal {T}_X$ lower semicontinuous. The same holds true also for the Tikhonov version with the alternative choice of $\alpha _k$ such that

$$\begin{aligned} \underline{\sigma }\le \frac{\Vert F'(x_k^\delta )(x_{k+1}^\delta (\alpha _k)-x_k^\delta )+F(x_k^\delta )-y^\delta \Vert }{\Vert F(x_k^\delta )-y^\delta \Vert }\le \overline{\sigma } \end{aligned}$$

for some constants $\underline{\sigma }$, $\overline{\sigma }$ satisfying $c_{tc}+\frac{1+c_{tc}}{\tau }<\underline{\sigma }<\overline{\sigma }<1$ in place of (11), as can be seen directly from (22). If $\mathcal {R}$ is defined by the norm on a space with the Kadets-Klee property, and $\mathcal {T}_X$ is the weak topology of this space, then this implies norm convergence of $x_{k_*(\delta ,y^\delta )}^\delta$ to $x^\dagger$ along the same subsequence.

### Remark 2

The fact that $x_k^\delta$ stays in $\mathcal {B}_R$ [cf. (15)] is crucial for the applicability of the tangential cone condition (8) in these iterates. If the functional $\mathcal {R}$ quantifies some distance to an a priori guess $x_0$, (e.g., $\mathcal {R}=\Vert x-x_0\Vert ^q$ for some norm $\Vert \cdot \Vert$ and some $q>0$), then $x\in \mathcal {B}_R$ with small *R* means closeness of *x* to $x_0$ in a certain sense. Thus, the smaller *R* is, the better (8) might get achievable with some $c_{tc}<\frac{1}{3}$. On the other hand, making *R* according to (15) small means closeness of $x^\dagger$ to $x_0$. Thus we deal with local convergence, as typical for Newton type methods.

Now we consider the appearance of discretization errors in the numerical solution of (5), (6) arising from restriction of the minimization to finite dimensional subspaces $X^k_h$ and leading to discretized iterates $x_{k,h}^\delta$ and an approximate version $F^k_h$ of the forward operator i.e., we consider the discretized version of Tikhonov-IRGNM (5)

31 $$\begin{aligned} x_{k+1,h}^\delta \in \mathrm{argmin}_{x\in \mathcal {C}\cap X^k_h} \ \Vert {F^k_h}'(x_{k,h}^\delta )(x-x_{k,h}^\delta )+F^k_h(x_{k,h}^\delta )-y^\delta \Vert ^p+\alpha _k\mathcal {R}(x), \end{aligned}$$

of Ivanov-IRGNM (6)

32 $$\begin{aligned} x_{k+1,h}^\delta \in \mathrm{argmin}_{x\in \mathcal {C}\cap X^k_h} \ \Vert {F^k_h}'(x_{k,h}^\delta )(x-x_{k,h}^\delta )+F^k_h(x_{k,h}^\delta )-y^\delta \Vert \text{ such } \text{ that } \mathcal {R}(x) \le \rho , \end{aligned}$$

and of Morozov-IRGNM (7)

33 $$\begin{aligned}&x_{k+1,h}^\delta \in \mathrm{argmin}_{x\in \mathcal {C}\cap X^k_h} \ \mathcal {R}(x) \text{ such } \text{ that } \Vert {F^k_h}'(x_{k,h}^\delta )(x-x_{k,h}^\delta )+F^k_h(x_{k,h}^\delta )-y^\delta \Vert \nonumber \\&\quad \le \sigma \Vert F^k_h(x_{k,h}^\delta )-y^\delta \Vert , \end{aligned}$$

respectively. Moreover, also in the discrepancy principle, the residual is replaced by its actually computable discretized version

34 $$\begin{aligned} k_*=k_*(\delta ,y^\delta )=\min \{k\in \mathbb {N}_0\ : \ \Vert F^k_h(x_{k,h}^{\delta })-y^\delta \Vert \le \tau \delta \}\,. \end{aligned}$$

**Fig. 1:**
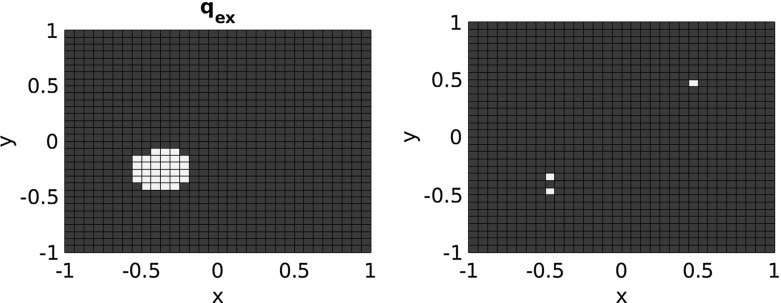
Left: exact source ${x}_{ex}$; right: locations of spots for testing weak * $L^\infty$ convergence

We define the auxiliary continuous iterates

35 $$\begin{aligned}&x_{k+1}^\delta \in \mathrm{argmin}_{x\in \mathcal {C}} \ \Vert F'(x_{k,h}^\delta )(x-x_{k,h}^\delta )+F(x_{k,h}^\delta )-y^\delta \Vert ^p+\alpha _k\mathcal {R}(x), \end{aligned}$$

36 $$\begin{aligned}&x_{k+1}^\delta \in \mathrm{argmin}_{x\in \mathcal {C}} \ \Vert F'(x_{k,h}^\delta )(x-x_{k,h}^\delta )+F(x_{k,h}^\delta )-y^\delta \Vert \text{ such } \text{ that } \mathcal {R}(x) \le \rho ,\nonumber \\ \end{aligned}$$

and

37 $$\begin{aligned}&x_{k+1}^\delta \in \mathrm{argmin}_{x\in \mathcal {C}} \ \mathcal {R}(x) \text{ such } \text{ that } \Vert F'(x_{k,h}^\delta )(x-x_{k,h}^\delta )+F(x_{k,h}^\delta )-y^\delta \Vert \nonumber \\&\quad \le \sigma \Vert F(x_{k,h}^\delta )-y^\delta \Vert , \end{aligned}$$

respectively in order to be able to use minimality, i.e., compare with the continuous exact solution $x^\dagger$. For an illustration we refer to [18, Figure 1].

First of all, we assess how large the discretization errors can be allowed to still enable convergence. Later on, in Sect. 3, we will describe how to really obtain such estimates a posteriori and to achieve the prescribed accuracy by adaptive discretization.

### Corollary 1

Let the assumptions of Theorem 1 be satisfied and assume that the discretization error estimates

38 $$\begin{aligned}&\Vert F(x_{k+1,h}^{\delta })-y^\delta \Vert -\Vert F(x_{k+1}^{\delta })-y^\delta \Vert \, \le \eta _{k+1} \end{aligned}$$

39 $$\begin{aligned}&\left| \Vert F_h^k(x_{k,h}^{\delta })-y^\delta \Vert -\Vert F(x_{k,h}^{\delta })-y^\delta \Vert \right| \, \le \xi _k \end{aligned}$$

40 $$\begin{aligned}&\mathcal {R}(x_{k,h}^{\delta })-\mathcal {R}(x_{k}^{\delta })\le \zeta _k \end{aligned}$$

(note that no absolute value is needed in (38), (40); moreover, (40) is only be needed for (5) and (7)) hold with

41 $$\begin{aligned} \eta _k\le c_\eta \Vert F_h^k(x_{k,h}^{\delta })-y^\delta \Vert \quad \xi _k\le c_\xi \Vert F_h^k(x_{k,h}^{\delta })-y^\delta \Vert , \quad \zeta _k\le \bar{\zeta }. \end{aligned}$$

for all $k\le k_*(\delta ,y^\delta )$ and constants $c_\eta ,c_\xi >0$ sufficiently small, $\bar{\zeta }>0$.

Then the assertions of Theorem 1 remain valid for $x^\delta _{k_*(\delta ,y^\delta ),h}$ in place of $x^\delta _{k_*(\delta ,y^\delta )}$ with (34) in place of (14) and (42) in place of (23).

### Proof

For the Tikhonov version (31), in order to inductively estimate $\mathcal {R}(x_{k+1,h}^\delta )$, given $x_k^\delta \in \mathcal {B}_R$, note that from (43) with $k+1$ replaced by *k*, we get like in (23) that

42 $$\begin{aligned} \mathcal {R}(x_{k+1,h}^\delta )\le & {} \left( \frac{1}{3}+\frac{4}{3\tau }\right) ^p \left( \frac{2}{3^p}-\frac{C}{(1-q)\tau ^p}\right) ^{-1}\nonumber \\&\times \left( 2^{1-p}\frac{\Vert F(x_0^\delta )-y^\delta \Vert ^p}{\alpha _0} +\frac{\mathcal {R}^\dagger +\bar{\zeta }}{\theta -q}\right) +\mathcal {R}^\dagger =:R \end{aligned}$$

where

$$\begin{aligned} d_{k,h}&:= 2^{1-p}(1-c_{tc})^p\Vert F(x_{k,h}^\delta )-y^\delta \Vert ^p,\\ \tilde{q}&:=2^{p-1}\bigl ((1+\gamma )^{p-1}+(1+\tilde{\gamma })^{p-1}\bigr )\left( \frac{c_{tc}}{1-c_{tc}}\right) ^{p}\,,\\ \quad q&=\tilde{q}+D\frac{c_\eta }{1-c_\xi }\ \in (0,1),\\ \mathcal {R}_{k,h}&:=\mathcal {R}(x_{k,h}^\delta ), \quad \mathcal {R}^\dagger :=\mathcal {R}(x^\dagger ),\\ C&:=\left( \frac{1+\gamma }{\gamma }\right) ^{p-1}(1+c_{tc})^p, \quad D:=\left( \frac{1+\tilde{\gamma }}{\tilde{\gamma }}\right) ^{p-1}(1-c_{tc})^p, \end{aligned}$$

for $\gamma , \tilde{\gamma }, c_\eta \in (0,1)$, which are chosen small enough so that $q<\theta$. As before, from the minimality of $x_{k+1}^\delta$ and (2), (8) as well as $x^\dagger \in \mathcal {D}(F)$, we have

$$\begin{aligned}&\Bigl ((1-c_{tc})\Vert F(x_{k+1}^\delta )-y^\delta \Vert -c_{tc}\Vert F(x_{k,h}^\delta )-y^\delta \Vert \Bigr )^p +\alpha _k\mathcal {R}(x_{k+1}^\delta ) \\&\quad \le \Bigl (c_{tc}\Vert F(x_{k,h}^\delta )-y^\delta \Vert +(1+c_{tc})\delta \Bigr )^p + \alpha _k\mathcal {R}(x^\dagger ), \end{aligned}$$

then using (38), (40),

$$\begin{aligned}&\left| (1-c_{tc})(\Vert F(x_{k+1,h}^\delta )-y^\delta \Vert -\eta _{k+1})-c_{tc}\Vert F(x_{k,h}^\delta )-y^\delta \Vert \right| ^p +\alpha _k\mathcal {R}(x_{k+1,h}^\delta ) \\&\quad \le \Bigl (c_{tc}\Vert F(x_{k,h}^\delta )-y^\delta \Vert +(1+c_{tc})\delta \Bigr )^p + \alpha _k\mathcal {R}(x^\dagger )+\alpha _k\zeta _{k+1}. \end{aligned}$$

Hence, with the same technique as in the proof of Theorem 1, using (24) with $\epsilon =\frac{1}{2}$, we have

$$\begin{aligned} d_{k+1,h}+\alpha _k \mathcal {R}_{k+1,h}\le & {} \tilde{q} d_{k,h} + \alpha _0\theta ^k (\mathcal {R}^\dagger +\zeta _{k+1})+C\delta ^p+D\eta _{k+1}^p\\\le & {} q d_{k,h} + \alpha _0\theta ^k (\mathcal {R}^\dagger +\zeta _{k+1})+ C \delta ^p\,, \end{aligned}$$

using (41). From this, by induction we conclude

43 $$\begin{aligned} d_{k+1,h}+\alpha _k\mathcal {R}_{k+1,h} \le q^{k+1}d_0 +\left( \frac{1}{1-\frac{q}{\theta }}\right) \alpha _k(\mathcal {R}^\dagger +\bar{\zeta })+\left( \frac{1}{1-q}\right) C \delta ^p \end{aligned}$$

Hence, by (39), (41), we have the following estimate

$$\begin{aligned}&\Vert F^k_h(x_{k,h}^\delta )-y^\delta \Vert \\&\quad \le \left( \delta +\left( \frac{2^{p-1}}{(1-c_{tc})^p} \left( \theta ^{k}\left( d_0+\frac{\alpha _0}{\theta -q}(\mathcal {R}^\dagger +\bar{\zeta })\right) + \frac{C}{1-q} \delta ^p\right) \right) ^{1/p}\right) \frac{1}{1-c_\xi }\,, \end{aligned}$$

where the right hand side falls below $\tau \delta$ as soon as

$$\begin{aligned} k&\ge (\log 1/\theta )^{-1}\left( p\log (1/\delta ) +\log \left( d_0+\frac{\alpha _0}{\theta -q}(\mathcal {R}^\dagger +\bar{\zeta })\right) -\log \left( \tilde{\tau }- \frac{C}{1-q} \right) \right) \\&=:\bar{k}(\delta ), \end{aligned}$$

for $\tilde{\tau }= 2^{1-p}(1-c_{tc})^p(\tau (1-c_\xi ))^p$. Note that $\tilde{\tau } > \frac{C}{1-q}$, provided $\tau$ is chosen sufficiently large, which we assume to be done. That is, we have shown that the discrepancy stopping criterion from (34) will be satisfied after finitely many, namely $O(\log (1/\delta ))$, steps.

On the other hand, the continuous discrepancy at the iterate defined by the discretized discrepancy principle (34) by (39), (41) satisfies

$$\begin{aligned} \Vert F(x_{k,h}^\delta )-y^\delta \Vert \le \tau (1+c_\xi )\delta \,. \end{aligned}$$

To estimate $\mathcal {R}(x^\delta _{k_*(\delta ,y^\delta ),h})$, note that according to our notation, from (43), we get, like in (23), that for all $k\in \{1,\ldots ,k_*(\delta ,y^\delta )\}$

$$\begin{aligned} \mathcal {R}_{k}\le & {} \theta \left( \frac{d_0}{\alpha _0}+\frac{\mathcal {R}^\dagger +\bar{\zeta }}{\theta -q}\right) \left( 1+ \frac{C}{1-q} \left( \tilde{\tau }- \frac{C}{1-q} \right) ^{-1}\right) =:R. \end{aligned}$$

Now we show finiteness of the stopping index for the discretized Ivanov-IRGNM (32). By minimality of $x_{k+1}^\delta$ and (38), for this problem we have

$$\begin{aligned} (1-c_{tc})\Vert F(x_{k+1,h}^\delta )-y^\delta \Vert \le 2c_{tc}\Vert F(x_{k,h}^\delta )-y^\delta \Vert +(1+c_{tc})\delta +(1-c_{tc})\eta _{k+1}. \end{aligned}$$

which with

$$\begin{aligned} d_{k,h}&:= (1-c_{tc})\Vert F(x_{k,h}^\delta )-y^\delta \Vert ,\\ \tilde{q}&:=\frac{2c_{tc}}{1-c_{tc}}\,, \quad q=\tilde{q}+D\frac{c_\eta }{1-c_\xi }\ \in (0,1),\\ C&:=(1+c_{tc}), \quad D:=(1-c_{tc}), \end{aligned}$$

by induction, (39) and (41) gives

$$\begin{aligned} \Vert F^k_h(x_{k,h}^\delta )-y^\delta \Vert \le \frac{1}{1-c_{tc}} d_{k,h} +\xi _{k} \le \frac{1}{1-c_{tc}}\left( q^{k}d_0 + \frac{C}{1-q} \delta \right) +\hat{\tau }\delta , \end{aligned}$$

where the right hand side is smaller than $\tau \delta$ for all

$$\begin{aligned} k \ge (\log 1/q)^{-1}\left( \log (1/\delta ) +\log d_0 -\log \left( \tilde{\tau }- \frac{C}{1-q} \right) \right) =:\bar{k}(\delta ), \end{aligned}$$

with $\tilde{\tau }=(1-c_{tc})\tau (1-c_\xi )$, so that we can again conclude $k_{*}(\delta ,y^\delta )\le \bar{k}(\delta )=O(\log (1/\delta ))$.

It remains to show finiteness of the stopping index for the discretized Morozov-IRGNM (33). By minimality of $x_{k+1}^\delta$ we have (30) with $x_k^\delta$ replaced by $x_{k,h}^\delta$, thus the inequalities (38) and (41) yield

$$\begin{aligned} \Vert F(x_{k+1,h}^\delta )-y^\delta \Vert \le \frac{\sigma +c_{tc}}{\left( 1-\frac{c_\eta }{1-c_\xi }\right) (1-c_{tc})}\Vert F(x_{k,h}^\delta )-y^\delta \Vert \end{aligned}$$

then, by (39) and induction

44 $$\begin{aligned} \Vert F^k_h(x_{k,h}^\delta )-y^\delta \Vert \le q^k \Vert F(x_{0,h}^\delta )-y^\delta \Vert \end{aligned}$$

where

$$\begin{aligned} q:=\frac{\sigma +c_{tc}}{\left( 1-\frac{c_\eta }{1-c_\xi }\right) (1-c_{tc})} \in (0,1), \end{aligned}$$

and the right hand side of (44) falls below $\tau \delta$ for all

$$\begin{aligned} k \ge (\log 1/q)^{-1}\left( \log (1/\delta ) +\log d_0 - \log \tilde{\tau } \right) =:\bar{k}(\delta ), \end{aligned}$$

where $\tilde{\tau }=\tau (1-c_\xi )$, and we can again conclude $k_{*}(\delta ,y^\delta )\le \bar{k}(\delta )=O(\log (1/\delta ))$.

Boundedness of the $\mathcal {R}$ values for (33) by $\mathcal {R}(x^\dagger )+\bar{\zeta }$ follows like in the proof of Theorem 1 together with (40), (41). $\square$

## Error estimators for adaptive discretization

The error estimators $\eta _k$, $\xi _k$ and $\zeta _k$ can be quantified, e.g., by means of a goal oriented dual weighted residual (DWR) approach [3], applied to the minimization problems

45 $$\begin{aligned} (x_{k+1,h}^\delta ,v_{k,h}^\delta ,u^\delta _{k+1},u_{k,h}^\delta )&\in \mathrm{argmin}_{(x,v,u,\tilde{u})\in \mathcal {C}\times V^3} \Vert C'(\tilde{u})v+C(\tilde{u})-y^\delta \Vert ^p+\alpha _k\mathcal {R}(x)\nonumber \\ \text{ s.t. } \forall w\in W:&\langle A'_x(x_{k,h}^\delta ,\tilde{u})(x-x_{k,h}^\delta )+A'_u(x_{k,h}^\delta ,\tilde{u})v,w\rangle _{W^*,W}=0,\nonumber \\&\langle A(x_{k,h}^\delta ,\tilde{u}),w\rangle _{W^*,W}=0, \quad \langle A(x,u),w\rangle _{W^*,W}=0,\nonumber \\ \end{aligned}$$

(note that the last constraint is added in order to enable computation of $I_2^k$ below)

46 $$\begin{aligned} (x_{k+1,h}^\delta ,v_{k,h}^\delta ,u^\delta _{k+1},u_{k,h}^\delta )&\in \mathrm{argmin}_{(x,v,u,\tilde{u})\in \mathcal {C}\times V^3} \frac{1}{2}\Vert C'(\tilde{u})v+C(\tilde{u})-y^\delta \Vert ^2\nonumber \\ \text{ s.t. }&\mathcal {R}(x)\le \rho _k,\nonumber \\ \text{ and } \forall w\in W:&\langle A'_x(x_{k,h}^\delta ,\tilde{u})(x-x_{k,h}^\delta )+A'_u(x_{k,h}^\delta ,\tilde{u})v,w\rangle _{W^*,W}=0,\nonumber \\&\langle A(x_{k,h}^\delta ,\tilde{u}),w\rangle _{W^*,W}=0, \quad \langle A(x,u),w\rangle _{W^*,W}=0,\nonumber \\ \end{aligned}$$

and

47 $$\begin{aligned} (x_{k+1,h}^\delta ,v_{k,h}^\delta ,u^\delta _{k+1},u_{k,h}^\delta )&\in \mathrm{argmin}_{(x,v,u,\tilde{u})\in \mathcal {C}\times V^3} \mathcal {R}(x)\nonumber \\ \text{ s.t. }&\Vert C'(\tilde{u})v+C(\tilde{u})-y^\delta \Vert \le \sigma \Vert C(\tilde{u})-y^\delta \Vert ,\nonumber \\ \text{ and } \forall w\in W:&\langle A'_x(x_{k,h}^\delta ,\tilde{u})(x-x_{k,h}^\delta )+A'_u(x_{k,h}^\delta ,\tilde{u})v,w\rangle _{W^*,W}=0,\nonumber \\&\langle A(x_{k,h}^\delta ,\tilde{u}),w\rangle _{W^*,W}=0, \quad \langle A(x,u),w\rangle _{W^*,W}=0,\nonumber \\ \end{aligned}$$

which are equivalent to (5), (6), and (7), respectively, with

$$\begin{aligned} I_1^k(x,v,u,\tilde{u})= & {} \Vert C(\tilde{u})-y^\delta \Vert \,, \quad I_2^k(x,v,u,\tilde{u})=\Vert C(u)-y^\delta \Vert \,,\quad \\ I_3^k(x,v,u,\tilde{u})= & {} \mathcal {R}(x) \end{aligned}$$

as quantities of interest [where $I_3^k$ is only needed for (5) and (7)]. We assume that $C,\mathcal {R}$ and the norms can be evaluated without discretization error, so the discretized versions of $I_i^k$ only arise due to discreteness of the arguments. Indeed, it is easy to see that the left hand sides of (38) and (39) can be bounded (at least approximately) by combinations of $I_1^k$ and $I_2^k$, using the triangle inequality:

48 $$\begin{aligned}&\Vert F(x_{k+1,h}^{\delta })-y^\delta \Vert -\Vert F(x_{k+1}^{\delta })-y^\delta \Vert \nonumber \\&\quad =I_1^{k+1}(x_{k+2}^\delta , v_{k+1}^\delta , u_{k+2}^\delta , \tilde{u}_{k+1}^\delta ) -I_1^{k+1}(x_{k+2,h}^\delta , v_{k+1,h}^\delta , u_{k+2,h}^\delta , \tilde{u}_{k+1,h}^\delta ) \nonumber \\&\qquad -\,(I_2^{k}(x_{k+1}^\delta , v_{k}^\delta , u_{k+1}^\delta , \tilde{u}_{k}^\delta ) -I_2^{k}(x_{k+1,h}^\delta , v_{k,h}^\delta , u_{k+1,h}^\delta , \tilde{u}_{k,h}^\delta )) +R_\eta ^{k+1}; \end{aligned}$$

49 $$\begin{aligned}&\Vert F_h^k(x_{k,h}^{\delta })-y^\delta \Vert -\Vert F(x_{k,h}^{\delta })-y^\delta \Vert \nonumber \\&\quad =I_1^{k}(x_{k+1,h}^\delta , v_{k,h}^\delta , u_{k+1,h}^\delta , \tilde{u}_{k,h}^\delta ) -I_1^{k}(x_{k+1}^\delta , v_{k}^\delta , u_{k+1}^\delta , \tilde{u}_{k}^\delta ) \,, \end{aligned}$$

where we will neglect $R_\eta ^{k+1}=\Vert F_h^{k+1}(x_{k+1,h}^{\delta })-y^\delta \Vert -\Vert F_h^k(x_{k+1,h}^{\delta })-y^\delta \Vert$.

It is important to note that $I_{1,h}^{k+1}$ is not equal to $I_{2,h}^k$, see [18].

The computation of the a posteriori error estimators $\eta _k, \xi _k, \zeta _k$ is done as in [18]. These error estimators can be used within the following adaptive algorithm for error control and mesh refinement: We start on a coarse mesh, solve the discretized optimization problem and evaluate the error estimator. Thereafter, we refine the current mesh using local information obtained from the error estimator, reducing the error with respect to the quantity of interest. This procedure is iterated until the value of the error estimator is below the given tolerance (41), cf. [3].

In this case, all the variables $x,v,u,\tilde{u}$ are subject to a new discretization. For better readability we will partially omit the iteration index *k* and the discretization index *h*. The previous iterate $x_k^\delta$ is fixed and not subject to a new discretization.

Consider now the cost functional for (45)

$$\begin{aligned} J(x,v,\tilde{u}) = \Vert C'(\tilde{u})v+C(\tilde{u})-y^\delta \Vert ^p+\alpha _k\mathcal {R}(x) \end{aligned}$$

and define the Langrangian functional

50 $$\begin{aligned} L(x,v,u,\tilde{u},\lambda ,\tilde{\mu },\mu ):=&J(x,v,\tilde{u})+\langle A'_x(x_k^\delta ,\tilde{u})(x-x_k^\delta )+A'_u(x_k^\delta ,\tilde{u})v,\lambda \rangle _{W^*,W}\nonumber \\&+\langle A(x_k^\delta ,\tilde{u}),\tilde{\mu }\rangle _{W^*,W}+\langle A(x,u),\mu \rangle _{W^*,W}\,, \end{aligned}$$

neglecting for simplicity (cf. Remark 2) the constraints defined by $\mathcal {C}$. The first-order necessary optimality conditions for (45) are given by stationarity for the Lagrangian *L*. Setting $z=(x,v,u,\tilde{u},\lambda ,\tilde{\mu },\mu )$, they read

$$\begin{aligned} L'(z)(dz)=0, \forall dz \in Z = X\times V\times V\times V\times W\times W\times W \end{aligned}$$

and for the discretized problem,

$$\begin{aligned} L'(z_h)(dz_h)=0, \forall dz_h \in Z_h = X_h\times V_h\times V_h\times V_h \times W_h\ \times W_h\times W_h\,. \end{aligned}$$

To derive a posteriori error estimators for the error with respect to the quantities of interest ($I_1, I_2, I_3$), we introduce auxiliary functionals $M_i$:

$$\begin{aligned} M_i(z,\bar{z})= I_i(z)+L'(z)\bar{z}, \quad z,\bar{z} \in Z, \quad i=1,2,3, \end{aligned}$$

Let $\tilde{z}=(z,\bar{z}) \in \tilde{Z} = Z \times Z$ and $\tilde{z}_h=(z_h,\bar{z}_h) \in \tilde{Z}_h = Z_h \times Z_h$ be continuous and discrete stationary points of $M_i$ satisfying

$$\begin{aligned} M'(\tilde{z})(d\tilde{z})=0, \forall d\tilde{z} \in Z \qquad M'(\tilde{z}_h)(d\tilde{z}_h)=0, \forall d\tilde{z}_h \in Z_h\,, \end{aligned}$$

respectively. Then, $z, z_h$ are continuous and discrete stationary points of *L* and there holds $I_i(z)=M_i(\tilde{z}), i = 1,2,3$. Thus the *z* part, as computed already during the numerical solution of the minimization problem (45) (or (46)) remains fixed for all $i\in \{1,2,3,\}$. Moreover, after computing the discrete stationary point $z_h$ for *L* (e.g., by applying Newton’s method), it requires only one more Newton step to compute the $\bar{z}$ coordinate of the stationary point for *M* from

$$\begin{aligned} L''(z_h)(\bar{z}_{i,h},d\bar{z})=-I_i'(z_h)d\bar{z} , \forall d\tilde{z}_h \in Z_h. \end{aligned}$$

According to [3], there holds

$$\begin{aligned} I_i(x,v,\tilde{u})-I_i(x_h,v_h,\tilde{u}_h)=\frac{1}{2}M'(\tilde{z}_h)(\tilde{z}-\hat{z}_h)+R, \quad \forall \hat{z}_h \in Z_h \quad i=1,2,3, \end{aligned}$$

with a remainder term *R* of order $O(\Vert \tilde{z}-\tilde{z}_h\Vert ^3)$ that is therefore neglected. Thus we use

$$\begin{aligned} I_i^k(z)-I_i^k(z_h) \approx \frac{1}{2}M_i'(z_h,\bar{z}_{i,h}) (\pi _h\tilde{z}_{i,h} - \tilde{z}_{i,h}) =\varepsilon ^k_i, \end{aligned}$$

where $\pi _h$ is an operator defined such that $(\pi _h\tilde{z}_{i,h} - \tilde{z}_{i,h})$ approximates the interpolation error as in [18], typically defined by local averaging, to define the estimators $\eta _k$, $\xi _k$, $\zeta _k$ according to the rule

51 $$\begin{aligned} \eta _{k+1}= \varepsilon ^{k+1}_1 +\varepsilon ^{k}_2\,, \quad \xi _k=\varepsilon ^{k}_1\,, \quad \zeta _k=\varepsilon ^{k}_3; \end{aligned}$$

cf. (48), (49). The estimators obtained by this procedure can be used to trigger local mesh refinement until the requirements (41) are met cf. [3].

Explictly, for $p=2$ (for simplicity) such a stationary point $z=(x,v,u,\tilde{u},\lambda ,\tilde{\mu })$ can be computed by solving the following system of equations (analogously for the discrete stationary point of *L*)

52 $$\begin{aligned}&-(A^{'}_x(x,u)^*\mu +A^{'}_{x} (x_k^\delta ,\tilde{u})^{*}\lambda ) \in \alpha _k \partial \mathcal {R}(x); \end{aligned}$$

53 $$\begin{aligned}&2\langle C^{'}(\tilde{u})(dv),C^{'}(\tilde{u})v+C(\tilde{u})-y^\delta \rangle +\langle A^{'}_u(x_k^\delta ,\tilde{u})(dv),\lambda \rangle =0, \quad \forall dv \in V; \qquad \end{aligned}$$

54 $$\begin{aligned}&\langle A^{'}_u(x,u)(du),\mu \rangle =0, \quad \forall du \in V; \end{aligned}$$

55 $$\begin{aligned}&\langle A^{''}_{xu} (x_k^\delta ,\tilde{u})(x-x_k^\delta ,d\tilde{u})+A^{''}_{uu} (x_k^\delta ,\tilde{u})(v,d\tilde{u}),\lambda \rangle +\langle A^{'}_u(x^\delta _k,\tilde{u})(d\tilde{u}),\tilde{\mu }\rangle \quad \quad \; \nonumber \\&\quad +\,2\langle C^{''}(\tilde{u})(d\tilde{u},v)+C^{'}(\tilde{u})(d\tilde{u}),C^{'}(\tilde{u})v+C(\tilde{u})-y^\delta \rangle =0, \quad \forall d\tilde{u} \in V; \end{aligned}$$

56 $$\begin{aligned}&\langle A^{'}_x (x_k^\delta ,\tilde{u})(x-x_k^\delta )+A^{'}_u (x_k^\delta ,\tilde{u})v,d\lambda \rangle = 0, \quad \forall d\lambda \in W; \end{aligned}$$

57 $$\begin{aligned}&\langle A(x_k^\delta ,\tilde{u}),d\tilde{\mu }\rangle =0, \quad \forall d\tilde{\mu } \in W; \end{aligned}$$

58 $$\begin{aligned}&\langle A(x,u),d\mu \rangle =0, \quad \forall d\mu \in W. \end{aligned}$$

Note that (58) is decoupled from the other equations and that if $A^{'}_u(x,u)^*$ is injective, Eq. (54) implies $\mu =0$.

Summarizing, since we have a convex minimization problem, after solving a nonlinear system of seven equations to find the minimizer, we need only one more Newton step to compute the error estimators to check whether we need a refinement on the mesh or not.

Regarding the problem (46) related to the Ivanov-IRGNM, we have the Lagrangian functional (50) with the cost functional defined by

$$\begin{aligned} J(x,v,\tilde{u}) = \frac{1}{2}\Vert C'(\tilde{u})v+C(\tilde{u})-y^\delta \Vert ^{2} +I_{(-\infty ,0]}(\mathcal {R}(x)-\rho )\,, \end{aligned}$$

and the indicator functional $I_{(-\infty ,0]}(\mathcal {R}(x)-\rho )$ takes the role of a regularization functional. The resulting optimality system is the same as above, cf. (52)-(58), just with (52) replaced by

59 $$\begin{aligned} -(A^{'}_x(x,u)^*\mu +A^{'}_{x} (x_k^\delta ,\tilde{u})^{*}\lambda ) \in \partial I_{(-\infty ,0]}(\mathcal {R}(x)-\rho ). \end{aligned}$$

Similarly for (47) for Morozov-IRGNM, with the cost function

$$\begin{aligned} J(x,v,\tilde{u}) = \mathcal {R}(x)+\underbrace{I_{(-\infty ,0]}\Bigl (\Vert C'(\tilde{u})v+C(\tilde{u})-y^\delta \Vert -\sigma \Vert C(\tilde{u})-y^\delta \Vert \Bigr )}_{=:\mathcal {Q}(\tilde{u},v)}\,, \end{aligned}$$

we end up with an optimality system by setting $\alpha _k=1$ and replacing (53), (55,56,57) in (52)–(58) by

60 $$\begin{aligned}&-A^{'}_u(x_k^\delta ,\tilde{u})^*\lambda \in \partial _v\mathcal {Q}(\tilde{u},v) \end{aligned}$$

61 $$\begin{aligned}&-(A^{''}_{xu} (x_k^\delta ,\tilde{u})(x-x_k^\delta )+A^{''}_{uu} (x_k^\delta ,\tilde{u})v)^*\lambda -A^{'}_u(x^\delta _k,\tilde{u})^*\tilde{\mu }\in \partial _{\tilde{u}}\mathcal {Q}(\tilde{u},v) \end{aligned}$$

respectively.

Note that the bound on $I_2$ only appears—via (51)—in connection to the assumption $\eta _k \le c_\eta \Vert F_h^k(x_{k,h}^{\delta })-y^\delta \Vert$, for $k \le k_*(\delta ,y^\delta )$ in (41). This may be satisfied in practice without refining explicitly with respect to $\eta _k$, but simply by refining with respect to the other error estimators $\xi _k$ (and $\zeta _k$ in the Tikhonov or Morozov case). The fact that $I^k_{1,h}$ and $I_{2,h}^{k-1}$ only differ in the discretization level, motivates the assumption that for small *h*, we have $I^k_{1,h} \approx I_{2,h}^{k-1}$ and $\eta _{k-1} \approx \xi _k$. Thefore, the algorithm used in actual computations will be built neglecting $I_2$ and hence skipping the constraint $\langle A(x,u),w\rangle _{W^*,W}=0, \;\forall w \in W$ in (45), (46), (47), which implies a modification of the Lagrangian (50) accordingly. Therefore, the corresponding optimality systems for $p=2$ in the Tikhonov case is given by

62 $$\begin{aligned}&-\,A^{'}_{x} (x_k^\delta ,\tilde{u})^{*}\lambda \in \alpha _k \partial \mathcal {R}(x); \end{aligned}$$

63 $$\begin{aligned}&2\langle C^{'}(\tilde{u})(dv),C^{'}(\tilde{u})v+C(\tilde{u})-y^\delta \rangle +\langle A^{'}_u(x_k^\delta ,\tilde{u})(dv),\lambda \rangle =0, \quad \forall dv \in V;\qquad \end{aligned}$$

64 $$\begin{aligned}&\langle A^{''}_{xu} (x_k^\delta ,\tilde{u})(x-x_k^\delta ,d\tilde{u})+A^{''}_{uu} (x_k^\delta ,\tilde{u})(v,d\tilde{u}),\lambda \rangle +\langle A^{'}_u(x^\delta _k,\tilde{u})(d\tilde{u}),\tilde{\mu }\rangle \quad \quad \; \nonumber \\&\quad +\,2\langle C^{''}(\tilde{u})(d\tilde{u},v)+C^{'}(\tilde{u})(d\tilde{u}),C^{'}(\tilde{u})v+C(\tilde{u})-y^\delta \rangle =0, \quad \forall d\tilde{u} \in V; \end{aligned}$$

65 $$\begin{aligned}&\langle A^{'}_x (x_k^\delta ,\tilde{u})(x-x_k^\delta )+A^{'}_u (x_k^\delta ,\tilde{u})v,d\lambda \rangle = 0, \quad \forall d\lambda \in W; \end{aligned}$$

66 $$\begin{aligned}&\langle A(x_k^\delta ,\tilde{u}),d\tilde{\mu }\rangle =0, \quad \forall d\tilde{\mu } \in W. \end{aligned}$$

Note that Eq. (66) is decoupled from the others. Therefore, the strategy is to solve (66) for $\tilde{u}$ first, then solve the linear system (62), (63), (65) for $(x,v,\lambda )$, and finally compute $\tilde{\mu }$ via the linear equation (64). Here, the system (62), (63), (65) can be interpreted as the optimality conditions for the following problem

$$\begin{aligned} (x_{k+1,h}^\delta ,v_{k,h}^\delta )&\in \mathrm{argmin}_{(x,v)\in \mathcal {C}\times V} \Vert C'(\tilde{u})v+C(\tilde{u})-y^\delta \Vert ^2+\alpha _k\mathcal {R}(x)\\ \text{ s.t. } \forall w\in W:&\langle A'_x(x_{k,h}^\delta ,\tilde{u})(x-x_{k,h}^\delta )+A'_u(x_{k,h}^\delta ,\tilde{u})v,w\rangle _{W^*,W}=0. \end{aligned}$$

For the Ivanov case, we have to solve (63)–(66) with

67 $$\begin{aligned} -A^{'}_{x} (x_k^\delta ,\tilde{u})^{*}\lambda \in \partial I_{(-\infty ,0]}(\mathcal {R}(x)-\rho ) \end{aligned}$$

in place of (62), hence again (66) is decoupled from the other equations, (64) is linear with respect to $\tilde{\mu }$, once $(x,v,\lambda )$ has been computed, and the remaining system for $(x,v,\lambda )$ can be interpreted as the optimality conditions for the following problem

$$\begin{aligned} (x_{k+1,h}^\delta ,v_{k,h}^\delta )&\in \mathrm{argmin}_{(x,v)\in \mathcal {C}\times V} \frac{1}{2}\Vert C'(\tilde{u})v+C(\tilde{u})-y^\delta \Vert ^2\\ \text{ s.t. }&\mathcal {R}(x)\le \rho _k,\\ \text{ and } \forall w\in W:&\langle A'_x(x_{k,h}^\delta ,\tilde{u})(x-x_{k,h}^\delta )+A'_u(x_{k,h}^\delta ,\tilde{u})v,w\rangle _{W^*,W}=0. \end{aligned}$$

The Morozov case requires solution of (62) (with $\alpha _k=1$), (65), (66), (60), (61). Thus again, we first solve (66) for $\tilde{u}$, then the system (62) (with $\alpha _k=1$), (65), (60), which is the first order optimality condition for

$$\begin{aligned} (x_{k+1,h}^\delta ,v_{k,h}^\delta )&\in \mathrm{argmin}_{(x,v)\in \mathcal {C}\times V} \mathcal {R}(x)\\ \text{ s.t. }&\Vert C'(\tilde{u})v+C(\tilde{u})-y^\delta \Vert \le \sigma \Vert C(\tilde{u})-y^\delta \Vert ,\\ \text{ and } \forall w\in W:&\langle A'_x(x_{k,h}^\delta ,\tilde{u})(x-x_{k,h}^\delta )+A'_u(x_{k,h}^\delta ,\tilde{u})v,w\rangle _{W^*,W}=0, \end{aligned}$$

with Lagrange multiplier $\lambda$ for the equality constraint, and finally the (now possibly nonlinear) inclusion (61) for $\tilde{\mu }$.

### Remark 3

Since DWR estimators are based on residuals which are computed in the optimization process, the additional costs for estimation are very low, which makes this approach attractive for our purposes. However, although these error estimators are known to work efficiently in practice (see [3]), they are not reliable, i.e., the conditions $I^k_i(z)-I^k_i(z_h) \le \epsilon _i^k$, $i=1,2,3$ can not be guaranteed in a strict sense in the computations, since we neglect the remainder term *R* and use an approximation for $\tilde{z}-\hat{z}_h$. As our analysis in Theorem 1 is kept rather general, it is not restricted to DWR estimators and would also work with different (e.g., reliable) error estimators.

## Model examples

We present a model example to illustrate the abstract setting from the previous section. Consider the following inverse source problem for a semilinear elliptic PDE, where the model and observation equations are given by

68 $$\begin{aligned} -\varDelta u +\kappa u^3= & {} \chi _{\omega _c}x \text{ in } \varOmega \subset \mathbb {R}^d, \end{aligned}$$

69 $$\begin{aligned} u= & {} 0 \text{ on } \partial \varOmega , \end{aligned}$$

70 $$\begin{aligned} C(u)= & {} u\mid _{\omega _o} , \ \Vert y-y^\delta \Vert _{L^2(\omega _o)} \le \delta , \end{aligned}$$

where $\chi _{\omega _c}$ denotes the extension by zero of a function on $\omega _c$ to a function on all of $\varOmega$. We first of all consider Tikhonov regularization and, aiming for a sparsely supported source, therefore use the space of Radon measures $\mathcal {M}(\omega _c)$ as a preimage space *X*. Thus we define the operators $A: \mathcal {M}(\omega _c)\times W^{1,q^{'}}_0 (\varOmega ) \longrightarrow W^{-1,q} (\varOmega )$, $A(x,u) = - \varDelta u + \kappa u^3 -\chi _{\omega _c}x$, $\kappa \in \mathbb {R}$ and the injection $C:W^{1,q^{'}}_0(\varOmega )\longrightarrow L^2(\omega _o)=Y$, $q > d$, where $\varOmega$ is a bounded domain in $\mathbb {R}^d$ with $d=2$ or 3, with Lipschitz boundary $\partial \varOmega$ and $\omega _c, \omega _o \subset \varOmega$ are the control domain and the observation domain, respectively.

A monotonicity argument yields well posedness of the above semilinear boundary value problem, i.e., well-definedness of $u\in W^{1,q^{'}}_0(\varOmega )$ as a solution to the elliptic boundary value problem (68), (69), as long as we can guarantee that $u^3\in W^{-1,q} (\varOmega )$ for any $u\in W^{1,q^{'}}_0(\varOmega )$, i.e., the embeddings $W^{1,q^{'}}_0(\varOmega )\rightarrow L^{3r}(\varOmega )$ and $L^r(\varOmega )\rightarrow W^{-1,q} (\varOmega )$ are continuous for some $r\in [1,\infty ]$, which (by duality) is the case iff $W^{1,q^{'}}_0(\varOmega )$ embeds continuously both into $L^{3r}(\varOmega )$ and $L^{r'}(\varOmega )$. By Sobolev’s Embedding Theorem, this boils down to the inequalities

$$\begin{aligned} 1-\frac{d}{q'}\ge -\frac{d}{3r} \text{ and } 1-\frac{d}{q'}\ge -\frac{d}{r'}\,, \end{aligned}$$

which by elementary computations turns out to be equivalent to

71 $$\begin{aligned} \frac{dq}{q+d}\le r \le \frac{dq}{3(dq-q-d)}\,, \end{aligned}$$

where the left hand side is larger than one and the denominator on the right hand side is positive due to the fact that for $d\ge 2$ we have $q>d\ge d'=\frac{d}{d-1}$. Taking the extremal bounds for $q>d$—note that the lower bound is increasing and the upper bound is decreasing with *q*—in (72) we get

72 $$\begin{aligned} \frac{d}{2}< r < \frac{d}{3(d-2)}\,. \end{aligned}$$

Thus, as a by-product, we get that for any $t\in [1,\bar{t})$ there exists $q>d$ such that $W^{1,q^{'}}_0(\varOmega )$ continuously embeds into $L^t$, with

73 $$\begin{aligned} \bar{t}=\infty \text{ in } \text{ case } d=2 \text{ and } \bar{t}=3 \text{ in } \text{ case } d=3\,. \end{aligned}$$

For the regularization functional $\mathcal {R}(x)=\Vert x\Vert _{\mathcal {M}(\omega _c)}$, the IRGNM-Tikhonov minimization step is given by (ignoring *h* in the notation)

$$\begin{aligned} (x_{k+1}^\delta ,v_{k}^\delta ,u_{k}^\delta )&\in \mathrm{argmin}_{(x,v,\tilde{u})\in \mathcal {M}(\omega _c)\times (W^{1,q^{'}}_0(\varOmega ))^2} \Vert v+\tilde{u}-y^\delta \Vert ^2_{L^2(\omega _o)} \\&\quad +\,\alpha _k\Vert x\Vert _{\mathcal {M}(\omega _c)}\\ \text{ s.t. } \forall w\in W^{1,q^{'}}_{0} (\varOmega ):&\int _{\varOmega }(\nabla v \nabla w+3\kappa \tilde{u}^2 vw)d\varOmega = \int _{\omega _c} wd(x-x_k^\delta ),\\&\int _{\varOmega } (\nabla \tilde{u}\nabla w+\kappa \tilde{u}^3w)d\varOmega = \int _{\omega _c} wdx_k^\delta . \end{aligned}$$

Here and below $\int _{\varOmega }\ d\varOmega$ and $\int _{\omega _c}\ dx$ denote the integrals with respect to the Lebesgue measure and with respect to the measure $x$, respectively.

Therefore, to compute this Gauss–Newton step, one first needs to solve the nonlinear equation

74 $$\begin{aligned} -\varDelta \tilde{u}+\kappa \tilde{u}^3=\chi _{\omega _c}x_k^\delta \end{aligned}$$

for $\tilde{u}=u_k^\delta$, then solve the following optimality system with respect to $(x,v,\lambda )$ (written in a strong formulation)

$$\begin{aligned}&\Vert \lambda \Vert _{C_b(\omega _c)} \le \alpha _k \text{ and } \int _\varOmega (x^{*} -\lambda )dx\le 0, \forall x^{*}\in B_{\alpha _k}^{C_b (\omega _c)}\\&\quad -\varDelta \lambda +3\kappa (u_k^\delta )^2\lambda +2v +2u_k^\delta =2\chi _{\omega _o}y^\delta \\&\quad -\varDelta v +3\kappa (u_k^\delta )^2v =\chi _{\omega _c}(x-x^\delta _k), \end{aligned}$$

which can be interpreted as the optimality system for the minimization problem

75 $$\begin{aligned} (x_{k+1}^\delta ,v_{k}^\delta )&\in \mathrm{argmin}_{(x,v)\in \mathcal {M}(\omega _c)\times W^{1,q^{'}}_0(\varOmega )} \Vert u_k^\delta +v-y^\delta \Vert ^2_{L^2(\omega _o)}+\alpha _k\Vert x\Vert _{\mathcal {M}(\omega _c)}\nonumber \\ \text{ s.t. }&-\varDelta v +3\kappa (u_k^\delta )^2 v = \chi _{\omega _c}(x-x^\delta _k), \end{aligned}$$

with Lagrange multiplier $\lambda$ for the equality constraint, and finally, compute $\tilde{\mu }$ by solving

76 $$\begin{aligned} -\varDelta \tilde{\mu } +3\kappa (u_k^\delta )^2\tilde{\mu }=-6\kappa u_k^\delta v\lambda -2(v+u_k^\delta -\chi _{\omega _o}y^\delta ). \end{aligned}$$

For carrying out the IRGNM iteration, $\tilde{\mu }$ is not required, but we need it for evaluating the error estimators.

For the Ivanov case, we consider the same model and observation equations (68), (69), (70) but now we intend to regularize by imposing $L^\infty$ bounds and thus use the slightly different function space setting, $A: L^\infty (\omega _c)\times H^{1}_0 (\varOmega ) \longrightarrow H^{-1} (\varOmega )$, $A(x,u) = - \varDelta u + \kappa u^3 -x$, $\kappa \in \mathbb {R}$ and the injection $C: H^1_0(\varOmega )\longrightarrow L^2(\omega _o)$.

The IRGNM-Ivanov minimization step with the regularization functional $\mathcal {R}(x)=\Vert x\Vert _{L^\infty (\omega _c)}$ is given by

$$\begin{aligned} (x_{k+1}^\delta ,v_{k}^\delta ,u_{k}^\delta )&\in \mathrm{argmin}_{(x,v,\tilde{u})\in L^\infty (\omega _c)\times (H^1_0(\varOmega ))^2} \Vert v+\tilde{u}-y^\delta \Vert ^2_{L^2(\omega _o)} \\ \text{ s.t. }&\Vert x\Vert _{L^\infty (\omega _c)} \le \rho \\ \text{ and } \forall w\in H^1_0 (\varOmega ):&\int _{\varOmega }(\nabla v \nabla w+3\kappa \tilde{u}^2 vw)d\varOmega = \int _{\omega _c} w(x-x_k^\delta )d\varOmega ,\\&\int _{\varOmega } (\nabla \tilde{u}\nabla w+\kappa \tilde{u}^3wd\varOmega = \int _{\omega _c} wx_k^\delta d\varOmega . \end{aligned}$$

For the Gauss–Newton step, one needs to first solve the nonlinear equation (74) for $\tilde{u}=u_k^\delta$, and then solve the following optimality system with respect to $(x,v,\lambda )$

$$\begin{aligned}&\Vert x\Vert _{L^{\infty }(\omega _c)} \le \rho \text{ and } \int _{\omega _c} (x^{*} -x)\lambda d\varOmega \le 0, \forall x^{*}\in B_{\rho }^{L^{\infty }(\omega _c)}\\&\quad -\varDelta \lambda +3\kappa (u_k^\delta )^2\lambda +2v +2u_k^\delta =2\chi _{\omega _o}y^\delta \\&\quad -\varDelta v +3\kappa (u_k^\delta )^2v =\chi _{\omega _c}(x-x^\delta _k), \end{aligned}$$

which can be interpreted as the optimality system for the minimization problem

77 $$\begin{aligned} (x_{k+1}^\delta ,v_{k}^\delta )&\in \mathrm{argmin}_{(x,v)\in L^{\infty }(\omega _c)\times H^1_0(\varOmega )} \frac{1}{2}\Vert u_k^\delta +v-y^\delta \Vert ^2_{L^2(\omega _o)}\nonumber \\ \text{ s.t. }&\Vert x\Vert _{L^\infty (\omega _c)} \le \rho \nonumber \\&-\varDelta v +3\kappa (u_k^\delta )^2 v = \chi _{\omega _c}(x-x^\delta _k) \end{aligned}$$

with Lagrange multiplier $\lambda$ for the equality constraint. Finally, $\tilde{\mu }$ is computed from (76).

For the IRGNM-Morozov case, using for simplicity the regularization functional $\mathcal {R}(x)=\frac{1}{2}\Vert x\Vert _{L^2(\omega _c)}^2$, and leaving the rest of the setting as in the IRGNM-Ivanov case, the step is defined by

$$\begin{aligned} (x_{k+1}^\delta ,v_{k}^\delta ,u_{k}^\delta )&\in \mathrm{argmin}_{(x,v,\tilde{u})\in L^\infty (\omega _c)\times (H^1_0(\varOmega ))^2} \frac{1}{2}\Vert x\Vert _{L^2(\omega _c)}^2 \\ \text{ s.t. }&\Vert v+\tilde{u}-y^\delta \Vert ^2_{L^2(\omega _o)}\le \sigma \Vert \tilde{u}-y^\delta \Vert ^2_{L^2(\omega _o)} \\ \text{ and } \forall w\in H^1_0 (\varOmega ):&\int _{\varOmega }(\nabla v \nabla w+3\kappa \tilde{u}^2 vw)d\varOmega = \int _{\omega _c} w(x-x_k^\delta )d\varOmega ,\\&\int _{\varOmega } (\nabla \tilde{u}\nabla w+\kappa \tilde{u}^3wd\varOmega = \int _{\omega _c} wx_k^\delta d\varOmega . \end{aligned}$$

So again we first solve (74) for $\tilde{u}=u_k^\delta$, then the minimization problem

$$\begin{aligned} (x_{k+1}^\delta ,v_{k}^\delta )&\in \mathrm{argmin}_{(x,v)\in L^\infty (\omega _c)\times H^1_0(\varOmega )} \frac{1}{2}\Vert x\Vert _{L^2(\omega _c)}^2 \\ \text{ s.t. }&\Vert v+u_k^\delta -y^\delta \Vert ^2_{L^2(\omega _o)}\le \sigma \Vert u_k^\delta -y^\delta \Vert ^2_{L^2(\omega _o)} \\&-\varDelta v +3\kappa (u_k^\delta )^2 v = \chi _{\omega _c}(x-x^\delta _k)\,, \end{aligned}$$

or actually its first order optimality system

$$\begin{aligned}&\lambda \vert _{\omega _c}=x\\&\phi \ge 0\,, \ \Vert v+u_k^\delta -y^\delta \Vert ^2_{L^2(\omega _o)}\le \sigma \Vert u_k^\delta -y^\delta \Vert ^2_{L^2(\omega _o)} \,, \\&\quad \phi \bigl (\Vert v+u_k^\delta -y^\delta \Vert ^2_{L^2(\omega _o)}-\sigma \Vert u_k^\delta -y^\delta \Vert ^2_{L^2(\omega _o)}\bigr )=0 \\&-\varDelta v +3\kappa (u_k^\delta )^2v =\chi _{\omega _c}(x-x^\delta _k), \end{aligned}$$

for $(x_{k+1}^\delta ,v_{k}^\delta ,\phi ,\lambda )$, and finally,

$$\begin{aligned} -\varDelta \tilde{\mu } +3\kappa (u_k^\delta )^2\tilde{\mu }=-6\kappa u_k^\delta v\lambda -\phi (v+(1-\sigma )(u_k^\delta -\chi _{\omega _o}y^\delta )). \end{aligned}$$

for $$\tilde{\mu }$$.

For numerically efficient methods to solve the minimization problems (75) and (77) we refer to e.g., [4–6] and the references therein.

We finally check the tangential cone condition in case $\omega _o=\varOmega$ and, for simplicity also $\omega _c=\varOmega$, in both settings

$$\begin{aligned} X=\mathcal {M}(\omega _c)\,, \quad V=W^{1,q'}_0(\varOmega )\,, \quad W=W^{1,q}_0(\varOmega ) \end{aligned}$$

(where we will have to restrict ourselves to $d=2$) and

$$\begin{aligned} X=L^\infty (\omega _c) \text{ or } X=L^2(\omega _c)\,, \quad V=W=H_0^1(\varOmega )\,. \end{aligned}$$

For this purpose, we use the fact that with the notation $F(\tilde{x})=\tilde{u}\vert _{\omega _o}$, $F(x)=u\vert _{\omega _o}$, $F(\tilde{x})-F(x)=v\vert _{\omega _o}$ and $F(\tilde{x})-F(x)-F'(x)(\tilde{x}-x)=w\vert _{\omega _o}$, the functions $v,w\in W^{1,q^{'}}_0(\varOmega )$ satisfy the homogeneous Dirichlet boundary value problems for the equations

$$\begin{aligned}&-\varDelta v+\kappa (\tilde{u}^2+\tilde{u}u+u^2)\, v =\tilde{x}-x\\&-\varDelta w+\kappa u^2 w = -\kappa (\tilde{u}+2u)\, v^2\,. \end{aligned}$$

Using an Aubin-Nitsche type duality trick, we can estimate the $L^2$ norm of *w* via the adjoint state $p\in W_0^{1,n}(\varOmega )$, which solves

$$\begin{aligned} -\varDelta p+\kappa u^2 p = w\,, \end{aligned}$$

with homogeneous Dirichlet boundary conditions, so that by Hölder’s inequality

$$\begin{aligned}&\Vert w\Vert _{L^2(\varOmega )}^2=\langle w,(-\varDelta +\kappa u^2\text{ id }) p\rangle =\langle (-\varDelta +\kappa u^2\text{ id })w, p\rangle \\&\quad = -\kappa \langle (\tilde{u}+2u)\, v^2, p\rangle \le \kappa \Vert v\Vert _{L^2(\varOmega )} \Vert \tilde{u}+2u\Vert _{L^m(\varOmega )} \Vert v\Vert _{L^m(\varOmega )} \Vert p\Vert _{L^{\frac{2m}{m-4}}(\varOmega )}\\&\quad \le \tilde{\tilde{C}} \kappa \Vert v\Vert _{L^2(\varOmega )} \Vert \tilde{u}+2u\Vert _{L^m(\varOmega )} \Vert v\Vert _{L^m(\varOmega )} \Vert w\Vert _{L^2(\varOmega )}\,, \end{aligned}$$

where we aim at choosing $m\in [4,\infty ]$, $n\in [1,\infty ]$ such that indeed

$$\begin{aligned} \Vert p\Vert _{W_0^{1,n}(\varOmega )}\le C \Vert w\Vert _{W^{-1,n}(\varOmega )}\le \tilde{C} \Vert w\Vert _{L^2(\varOmega )} \end{aligned}$$

and the embeddings $V\rightarrow L^m(\varOmega )$, $W^{1,n}(\varOmega )\rightarrow L^{\frac{2m}{m-4}}(\varOmega )$, $L^2(\varOmega )\rightarrow W^{-1,n}(\varOmega )$ are continuous. If we succeed in doing so, we can bound $\tilde{\tilde{C}} \kappa \Vert \tilde{u}+2u\Vert _{L^m(\varOmega )} \Vert v\Vert _{L^m(\varOmega )}$ by some constant $c_{tc}$, which will be small provided $\Vert \tilde{x}-x\Vert _X$ and hence $\Vert v\Vert _{L^m(\varOmega )}$ is small. Thus, the numbers *n*, *m* are limited by the requirements

78 $$\begin{aligned} V\subseteq L^m(\varOmega ) \text{ and } W^{1,n}(\varOmega )\subseteq L^{\frac{2m}{m-4}}(\varOmega ) \text{ and } m\ge 4\,, \end{aligned}$$

$$L^2(\varOmega )\subseteq W^{-1,n}(\varOmega )$$, i.e., by duality,

79 $$\begin{aligned} W_0^{1,n'}(\varOmega )\subseteq L^2(\varOmega )\,, \end{aligned}$$

and the fact that $\kappa u^2 p\in L^o(\varOmega )$ should be contained in $W^{-1,n'}(\varOmega )$ for $u\in V\subseteq L^t(\varOmega )$, and $p\in W^{1,n}(\varOmega )$, which via Hölder’s inequality in

$$\begin{aligned} \left( \int _\varOmega (u^2p)^o\, d\varOmega \right) ^{1/o} \le \Vert u\Vert _{L^t(\varOmega )}^2 \Vert p\Vert _{L^\frac{ot}{t-2o}(\varOmega )} \end{aligned}$$

and duality leads to the requirements

80 $$\begin{aligned} W_0^{1,n}(\varOmega )\subseteq L^{o'}(\varOmega ) \text{ and } V\subseteq L^t(\varOmega ) \text{ and } W_0^{1,n}(\varOmega )\subseteq L^\frac{ot}{t-2o}(\varOmega ) \text{ and } o\le \frac{t}{2} \end{aligned}$$

In case $V=W_0^{1,q'}(\varOmega )$ with $q>d$ and $d=3$, (78) will not work out, since according to (73), *m* cannot be chosen larger or equal to four.

In case $V=W_0^{1,q'}(\varOmega )$ with $q>d$ and $d=2$, we can choose, e.g., $t=m=n=6$, $o=2$ to satisfy (78), (79), (80) as well as $t,m<\bar{t}$ as in (73).

The same choice is possible in case $V=H_0^1(\varOmega )$ with $d\in \{2,3\}$.

## Numerical tests

In this section, we provide some numerical illustration of the IRGNM Ivanov method applied to the example from Sect. 4, i.e., each Newton step consists of solving (74) and subsequently (77). For the numerical solution of (74) we apply a damped Newton iteration to the equation $\varPhi (\tilde{u})=0$ where

$$\begin{aligned}&\varPhi : H_0^1(\varOmega )\rightarrow H^{-1}(\varOmega )\,,\quad \varPhi (\tilde{u})=-\varDelta \tilde{u}+\kappa \tilde{u}^3-x^\delta _k\,,\\&\tilde{u}^{l+1}=\tilde{u}^{l}-\Bigl (-\varDelta \tilde{u}+3\kappa (\tilde{u}^l)^2\Bigr )^{-1}\Bigl (-\varDelta \tilde{u}+\kappa (\tilde{u}^l)^3-x_k^\delta \Bigr )\,, \end{aligned}$$

which is stopped as soon as $\Vert \varPhi (\tilde{u}^l)\Vert _{H^{-1}(\varOmega )}$ has been reduced by a factor of 1.e−4. The sources $x$ and states *u* are discretized by piecewise linear finite elements, hence after elimination of the state via the linear equality constraint, (77) becomes a box constrained quadratic program for the dicretized version of $x$, which we solve with the method from [12] using the Matlab code mkr_box provided to us by Philipp Hungerländer, Alpen-Adria Universität Klagenfurt. All implementations have been done in Matlab.

We performed test computations on a 2-d domain $\omega _o=\omega _c=\varOmega =(-1,1)^2$, on a regular computational finite element grid consisting of $2\cdot N\cdot N$ triangles, with $N=32$. We first of all consider $\kappa =1$ (below we will also show results with $\kappa =100$) and the piecewise constant exact source function

81 $$\begin{aligned} {x}_{ex}=-10+20\cdot {\mathbb {1}}_{B}\,, \end{aligned}$$

where *B* is the ball of radius 0.2 around $(-0.4,-0.3)$ cf. Fig. 1, and correspondingly set $\rho =10$. In order to avoid an inverse crime, we generated the synthetic data on a finer grid and, after projection of $u_{ex}$ onto the computational grid, we added normally distributed random noise of levels $\delta \in \{0.001, 0.01, 0.1\}$ to obtain synthetic data $y^\delta$.

In all tests we start with the constant function with value zero for $x_0$. Moreover, we always set $\tau =1.1$. According to our convergence result Theorem 1 with $\mathcal {R}=\Vert \cdot \Vert _{L^\infty (\varOmega )}$, we can expect weak * convergence in $L^\infty (\varOmega )$ here. Thus we computed the errors in certain spots within the two homogeneous regions and on their interface,

$$\begin{aligned} \text{ spot }_1=(0.5,0.5)\,, \quad \text{ spot }_2=(-0.4,-0.3)\,, \quad \text{ spot }_3=(-0.4,-0.5)\,, \quad \end{aligned}$$

cf. Fig. 1, more precisely, on $\frac{1}{N}\times \frac{1}{N}$ squares located at these spots, corresponding to the piecewise constant $L^1$ functions with these supports in order to exemplarily test weak * $L^\infty$ convergence. Additionally we computed $L^1$ errors.

Table 1 provides an illustration of convergence as $\delta$ decreases. For this purpose, we performed five runs on each noise level for each example and list the average errors.

**Table 1:** Convergence as $\delta \rightarrow 0$: averaged errors of five test runs with uniform noise

$\delta$	$\text{ err }_{spot_1}$	$\text{ err }_{spot_2}$	$\text{ err }_{spot_3}$	$\text{ err }_{L^1(\varOmega )}$
0.1000	0	4.0818	8.0043	0.0627
0.0667	0.1558	3.6454	7.8451	0.0541
0.0333	0	3.0442	6.5726	0.0370
0.0100	0	0	3.9091	0.0188

**Fig. 2:**
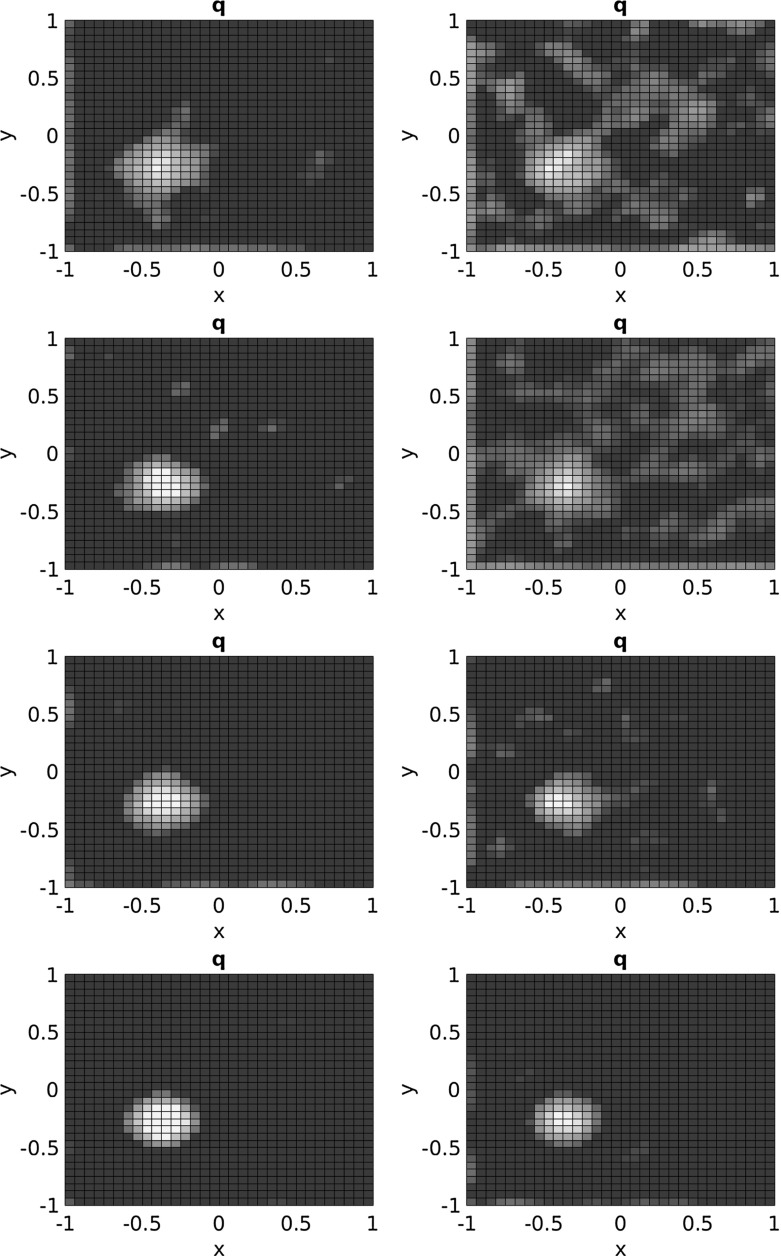
Reconstructions from noisy data with $\delta \in \{0.1, 0.667,0.333,0.01\}$ (top to bottom) for $\kappa =1$ (left) and $\kappa =100$ (right)

**Fig. 3:**
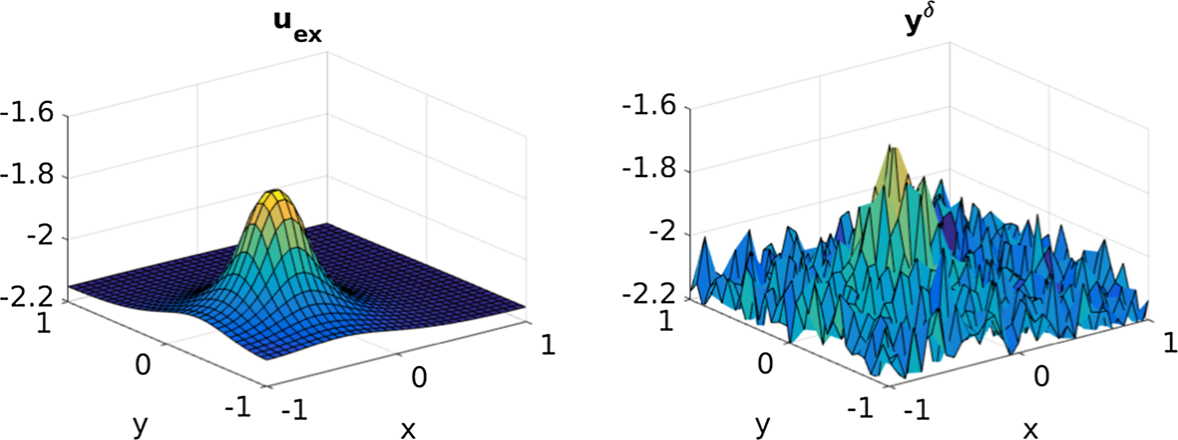
Exact and noisy data ($\delta =0.1$) for $\kappa =1$

**Fig. 4:**
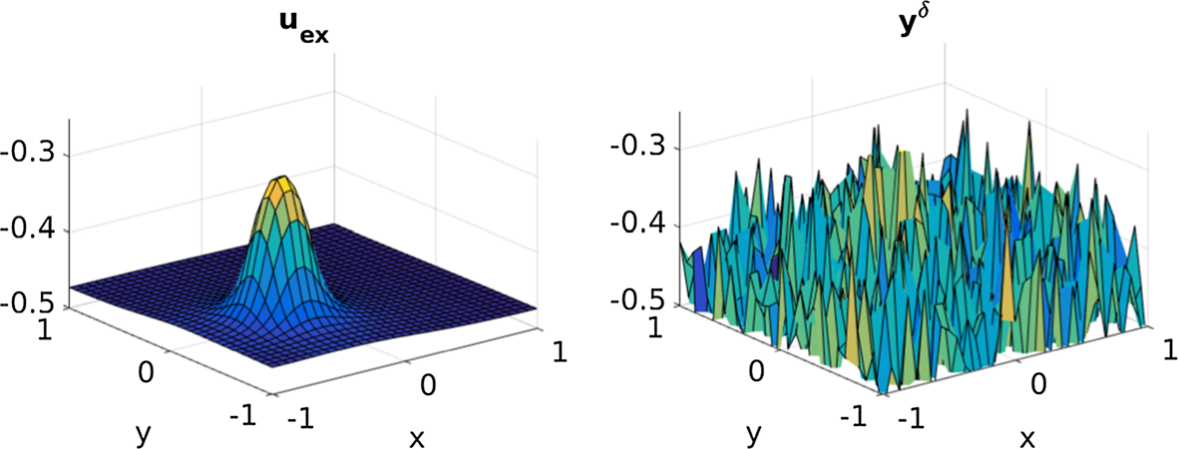
Exact and noisy data ($\delta =0.1$) for $\kappa =100$

In Fig. 2 we plot the reconstructions for $\kappa =1$ and $\kappa =100$. For $\kappa =1$, the noise levels $\delta \in \{0.1, 0.667,0.333,0.01\}$ correspond to a percentage of $p\in \{5.6, 18.5, 37.1, 55.6\}$ of the $L^2$ deviation of the exact state from the background state $u_0=-10^{1/3}$. In case of $\kappa =100$, where the background state is $u_0=-0.1^{1/3}$ the corresponding percentages are $p\in \{17.9,59.7,119.4,179.2\}$. For an illustration of the noisy data as compared to the exact ones, see Figs. 3 and 4. Indeed, the box constraints enable to cope with relatively large noise levels, even in the rather nonlinear regime with $\kappa =100$.

## Conclusions and remarks

In this paper we have studied convergence of the Tikhonov type, the Ivanov type, and the Morozov type IRGNM with a stopping rule based on the discrepancy principle type. To the best of our knowledge, the Ivanov and Morozov IRGNMs have not been studied so far and in all three Tikhonov, Ivanov, and Morozov type IRGNMs, convergence results without source conditions so far use stronger assumptions than the tangential cone condition used here. We also consider discretized versions of the methods and provide discretization error bounds that still guarantee convergence. Moroever, we discuss goal oriented dual weighted residual error estimators that can be used in an adaptive discretization scheme for controlling these discretization error bounds. An inverse source problem for a nonlinear elliptic boundary value problems illustrates our theoretical findings in the special situations of measure valued and $L^\infty$ sources. We also provide some computational results with the Ivanov IRGNM for the case of an $L^\infty$ source. Numerical implementations and tests for a measure valued source, together with adaptive discretization is subject of ongoing work, based on the approaches from [4–6, 18, 19]. Future research in this context will be concerend with convergence rates results for the Ivanov and Morozov IRGNMs under source conditions.

## Appendix

### Lemma 1

For all $a,b > 0,$ $p \ge 1$ and $\gamma , \epsilon \in (0,1)$

82 $$\begin{aligned} (a+b)^p \le (1+\gamma )^{p-1}a^p+\left( \frac{1+\gamma }{\gamma }\right) ^{p-1}b^p \end{aligned}$$

and, if additionally $a\ge b$, also

83 $$\begin{aligned} (a-b)^p \ge (1-\epsilon )^{p-1}a^p-\left( \frac{1-\epsilon }{\epsilon }\right) ^{p-1}b^p. \end{aligned}$$

### Proof

The estimate in (82) can be done by solving the following extremal value problems

$$\begin{aligned} C_\gamma = \max _{x>0}{\phi (x)}\,, \quad C_\epsilon = \max _{x>0}{\varPhi (x)}\,, \end{aligned}$$

where

$$\begin{aligned} \phi (x):= ((1+x)^p-(1+\gamma )^{p-1})x^{-p} \text{ and } \varPhi (x):= ((1-\epsilon )^{p-1}-(1-x)^p)x^{-p}, \end{aligned}$$

since for any $\gamma ,\epsilon \in (0,1)$,

$$\begin{aligned} \phi (x)\le C_\gamma \text{ and } \varPhi (x)\le C_\epsilon \text{ for } \text{ all } x>0 \end{aligned}$$

with $x:=b/a$, $a,b>0$ is equivalent to (24).

Solving for $C_\gamma$, we have

$$\begin{aligned} \phi ' (x) = px^{-(p+1)}((1+\gamma )^{p-1}-(1+x)^{p-1})\left\{ \begin{array}{ll} =0 &{}\Longleftrightarrow x=\gamma ,\\<0 &{} \text{ for } x>\gamma ,\\ >0 &{} \text{ for } x<\gamma , \end{array}\right. \end{aligned}$$

which means that

$$\begin{aligned} \max \phi (x)=\phi (\gamma )=\left( \frac{1+\gamma }{\gamma }\right) ^{p-1}, \end{aligned}$$

so defining $C_\gamma :=\left( \frac{1+\gamma }{\gamma }\right) ^{p-1}$ and writing the resulting inequality in terms of *a* and *b* we have the desired formula.

The formula in (83) is derived analogously. $\square$

### Lemma 2

Let $$k\in \mathbb {N}$$, $$(d_j)_{1\le j\le k+1}\subseteq [0,\infty )$$, $$(\mathcal {R}_j)_{1\le j\le k+1}\subseteq [0,\infty )$$, $$\alpha _0,\mathcal {R}^\dagger ,c,q\not =\theta \in (0,\infty )$$. Then

84 $$\begin{aligned} \forall j\in \{0,\ldots ,k\}\, : \ d_{j+1}+\alpha _0\theta ^j\mathcal {R}_{j+1}\le qd_j +\alpha _0\theta ^j\mathcal {R}^\dagger +c\,, \end{aligned}$$

implies that

85 $$\begin{aligned} d_{k+1}+\alpha _0\theta ^k\mathcal {R}_{k+1} < q^{k+1}d_0 +\left( \frac{1}{1-\frac{q}{\theta }}\right) \alpha _0\theta ^k\mathcal {R}^\dagger +\left( \frac{1}{1-q}\right) c. \end{aligned}$$

### Proof

We first show by induction that for all $l\in \{0,\ldots ,k\}$

86 $$\begin{aligned} d_{k+1}+\alpha _0\theta ^k\mathcal {R}_{k+1} \le q^{l+1} d_{k-l}+\left( 1+\frac{q}{\theta }+\cdots +\left( \frac{q}{\theta }\right) ^l\right) \alpha _0 \theta ^k\mathcal {R}^\dagger +(1+q+\cdots +q^l)c. \end{aligned}$$

Indeed, for $l=0$, (86) is just (84) with $j=k$. Suppose that (86) holds for *l*, then using (84) with $j=k-(l+1)$, we obtain the formula for $l+1$

$$\begin{aligned}&d_{k+1}+\alpha _k\mathcal {R}_{k+1} \\&\quad \le q^{l+1}\Bigl (qd_{k-(l+1)}+\alpha _0 \theta ^{k-(l+1)}\mathcal {R}^\dagger +c\Bigr )\\&\qquad +\left( 1+\frac{q}{\theta }+\cdots +\left( \frac{q}{\theta }\right) ^l\right) \alpha _0 \theta ^k\mathcal {R}^\dagger +(1+q+\cdots +q^l)c\\&\quad = q^{(l+1)+1} d_{k-(l+1)}+\left( 1+\frac{q}{\theta }+\cdots +\left( \frac{q}{\theta }\right) ^l+\left( \frac{q}{\theta }\right) ^{l+1}\right) \alpha _0 \theta ^k\mathcal {R}^\dagger \\&\qquad +\,(1+q+\cdots +q^l+q^{l+1})c, \end{aligned}$$

and the induction proof is complete.

Hence, setting $l=k$ in (86) and using the geometric series formula, we get the assertion (85).
